# Neuroimaging correlates of post-stroke fatigue: A systematic review and meta-analysis

**DOI:** 10.1177/17474930231192214

**Published:** 2023-08-12

**Authors:** Amy A Jolly, Adriana Zainurin, Gillian Mead, Hugh S Markus

**Affiliations:** 1 Stroke Research Group, Department of Clinical Neurosciences, University of Cambridge, Cambridge Biomedical Campus, Cambridge, UK; 2 Usher Institute, The University of Edinburgh, Edinburgh, UK

**Keywords:** Stroke, fatigue, MRI, neuroimaging, post-stroke fatigue

## Abstract

**Background:**

Fatigue is a common and disabling symptom following stroke, but its underlying mechanisms are unknown. Associations with a number of imaging features have been proposed.

**Aims:**

We aimed to assess whether neuroimaging parameters could better inform our understanding of possible causes of post-stroke fatigue (PSF) through systematic review and meta-analysis.

**Methods:**

Using a predefined protocol registered with PROSPERO (ID: CRD42022303168), we searched EMBASE, MEDLINE, PubMed, and PsycInfo for studies assessing PSF and computerized tomography (CT), magnetic resonance (MR), positron emission tomography (PET) imaging, or diffusion tensor imaging (DTI). We extracted neuroimaging parameters and narratively analyzed study results to assess any association with PSF. Where there were 3+ similar studies, we carried out a meta-analysis using inverse-variance random-effects model to estimate the total association of each neuroimaging parameter on PSF. The risk of bias was assessed using the Newcastle and Ottawa Scale.

**Results:**

We identified 46 studies (*N* = 6543); in many studies, associations with fatigue were secondary or subanalyses (28.3%). Imaging parameters were assessed across eight variables: lesion lateralization, lesion location, lesion volume, brain atrophy, infarct number, cerebral microbleeds, white matter hyperintensities (WMHs), and network measures. Most variables showed no conclusive evidence for any association with fatigue. Meta-analysis, where possible, showed no association of the following with PSF; left lesion lateralization (OR: 0.88, 95% CI (0.64, 1. 22) (*p* *=* 0.45)), infratentorial lesion location (OR: 1.83, 95% CI (0.63, 5.32) (*p* *=* 0.27)), and WMH (OR: 1.21, 95% CI (0.84, 1.75) (*p* *=* 0.29)). Many studies assessed lesion location with mixed findings; only one used voxel-symptom lesion-mapping (VSLM). Some small studies suggested an association between altered functional brain networks, namely frontal, fronto-striato-thalamic, and sensory processing networks, with PSF.

**Conclusion:**

There was little evidence for the association between any neuroimaging parameters and PSF. Future studies should utilize advanced imaging techniques to fully understand the role of lesion location in PSF, while the role of altered brain networks in mediating PSF merits further research.

## Introduction

Fatigue is a common and often debilitating symptom following both ischemic and hemorrhagic stroke. It has a major impact on quality of life^1,2^ and has been identified as one of the unmet needs in stroke research.^3^ Depending on the definition used, estimates of the prevalence of post-stroke fatigue (PSF) are around 50%.^4,5^ Despite its importance, there are no proven treatments for PSF, a fact partly explained by a lack of understanding of its underlying pathophysiology. Potential suggested mechanisms include altered cortical excitability, involvement of specific brain regions, disruption of complex brain networks, and systemic inflammation.^6^

If the involvement of specific brain regions or altered brain network activity does play a role, then one might expect neuroimaging to provide important insights into the pathophysiology of PSF. This includes identifying associations with structural anatomical features such as infarct location and size, as well as associations with network integrity and “brain activity.” Most studies describing associations of neuroimaging features with fatigue have been small, and while some have reported associations, for example, with white matter hyperintensity (WMH),^7^ and subcortical infarcts^7,8^ others have not replicated these.^1,9^

To better understand the relationship between brain structure and function and fatigue and gain insights into the pathophysiology of PSF, we performed a systematic review of neuroimaging variables and their relationship to PSF.

## Methods

In line with PRISMA guidelines,^10^ the systematic review protocol was preregistered on PROSPERO (ID: CRD42022303168).^11^

### Searches

We searched four electronic databases using our predefined search strategy (available in Supplemental Appendix 1)) on 20 May 2023. These databases were: EMBASE (from 1974), MEDLINE (from 1946), PubMed (from 1950), and PsycInfo (from 1955). Studies had to assess fatigue and stroke and neuroimaging to be included in record screening (see search terms, Supplemental Appendix 1).

### Inclusion and exclusion criteria

We included randomized controlled trials, observational, cohort, case–control, and cross-sectional studies. Only studies in the English language were included.

Studies were included if they assessed stroke patients over 16years old, with any subtype of stroke (e.g., ischemic or intracranial hemorrhage), and any duration since stroke. Studies had to include any assessment of fatigue and at least one of the following imaging modalities: computerized tomography (CT), magnetic resonance (MR), positron emission tomography (PET) imaging, or diffusion tensor imaging (DTI).

Studies were excluded if they assessed patients: under 16, included transient ischemic attack or subarachnoid hemorrhage, or did not assess any of the variables listed above. Gray literature, unpublished literature, abstracts, narrative reviews, and case reports and series in <5 subjects were excluded.

### Data extraction

Two reviewers (A.A.J. and A.Z.) independently screened all titles and abstracts. Any disagreement between reviewers was resolved by discussion between the two reviewers and, if no consensus was obtained, by discussion with a third reviewer (H.S.M.).

### Risk of bias

The Newcastle and Ottawa scale (NOS)^12^ was utilized to assess the risk of bias. We used the original NOS^12^ and the modified NOS for cross-sectional research^13^ (Supplementary sTable 1). There are no set cut-offs, but higher scores indicate higher-quality studies.^14^

### Data synthesis

As this was an exploratory review, we planned to look narratively at studies that corrected for confounders and those that did not together. Main findings for each outcome measure were narratively described. Where appropriate, if three or more studies were sufficiently similar, in both fatigue measure used and neuroimaging parameter, meta-analysis was performed with R (version 4.2.2) package metafor (v3.8-1),^15^ using an inverse-variance random-effects model, to estimate the total effect of each neuroimaging measure on PSF. To retain the maximum number of studies in our meta-analyses, we included studies binarizing fatigue using validated cut-offs across multiple measures. We included only raw data or univariate odds ratios in our meta-analyses to ensure consistency in included studies. A *p* value of 0.05 was used to determine statistical significance.

## Results

### Identified studies

**Figure 1.**
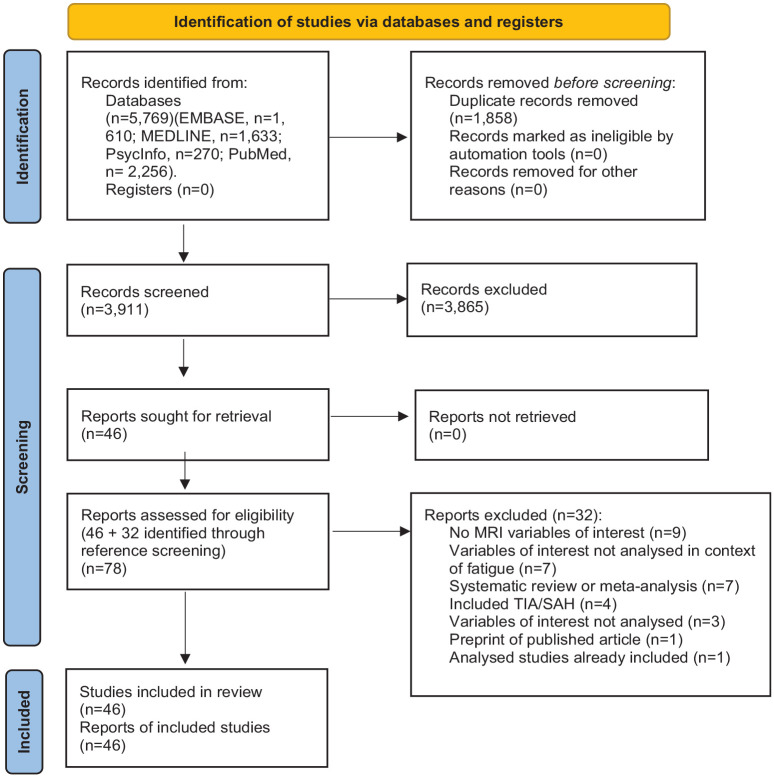
PRISMA flow diagram^10^ showing the selection process.

Our search strategy identified 5769 records; 1858 were duplicates and were removed. Of the remaining 3911, through title and abstract screening, we identified 46 records that met inclusion criteria. The most common reasons for exclusion were the wrong study population (*n* = 3026), not assessing fatigue (*n* = 2659), and case studies (*n* = 976). Most excluded studies met multiple exclusion criteria. Searching reference lists identified a further 32 records, resulting in 78 studies for the full-text screen. All full texts were obtained. Of these, 32 records were excluded (Figure 1). In the case of systematic reviews and meta-analyses, we extracted the studies and included them if they fulfilled the inclusion criteria. Based on neuroimaging parameters reported, the remaining 46 studies were divided into 8 subcategories (Table 1). Many studies assessed multiple neuroimaging variables.

**Table 1.** Neuroimaging variable categories and *N* of studies assessing variables.

Neuroimaging category	*N* of studies	*N* of participants	References
Lesion lateralization	29	3709	^9,16–43^
Lesion location	19	3036	^1,7–9,19,27,29,31,33,38,43–51^
Lesion volume	16	2755	^1,7,20,21,31,32,35,43–49,52,53^
Brain atrophy	4	540	^1,7,9,39^
Number of infarcts	6	1455	^1,39,44–47^
Cerebral microbleeds	1	199	^46^
WMHs	7	1336	^1,7,9,39,44–46^
Network measures	8	334	^21,35,38,52,54–57^

WMH: white matter hyperintensity.

The 46 studies comprised a total of 6543 participants. The most common scale used to assess fatigue was the Fatigue Severity Scale (FSS) (*n* = 20 studies), and the second most common was the Multidimensional Fatigue Inventory (MFI) (*n* = 8). We noted that many studies assessed fatigue as a single construct. Time from stroke to fatigue assessment ranged from the acute stage (<7 days) to 7+ years; the most common timepoint was 3 months post-stroke (*n* = 18 studies). Demographics and details of the 46 individual studies are shown in Supplementary sTable 2.

### Associations of PSF with specific neuroimaging variables

#### Lesion lateralization

Twenty-nine studies (*n* = 3709 participants) assessed whether lesion lateralization was associated with PSF.^9,16–43^ Of these, 24 showed no association, 3 reported a higher prevalence of PSF with right-sided stroke,^29,34,38^ and 1 showed a higher prevalence of PSF in right-sided stroke at 6-month follow-up but not at baseline.^43^ One study reported higher rates of general and mental fatigue in those with left-sided lesion lateralization.^42^

Sixteen studies had group data or summary data suitable for meta-analysis.^9,16,17,19,21,24,25,27,28,32,34,37,38,43,52,54^ Two studies had data for both baseline and follow-up,^9,43^ we used only the earliest timepoint to remain consistent with the other studies. One study looked at fatigue across three groups (low, moderate, and severe). We collapsed these into two groups: low-moderate (FSS score < 4) versus severe (FSS score > 4), in line with other studies.^25^ One study had data presented as percentages for two measures; we only included the measure for which percentages could be converted to the total sample size.^34^

**Figure 2.**
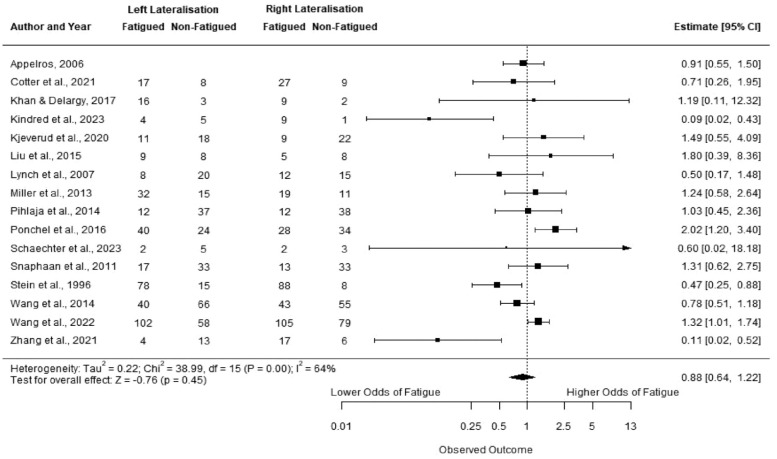
Forest plot showing the association between lesion lateralization (left-sided stroke) and prevalence of PSF. Liu et al.^27^ and Kjeverud et al.^25^ compare low-moderate vs severe fatigue. For Pihlaja et al.,^32^ Snaphaan et al.^9^ and Stein et al.^34^ group data were calculated using percentages.

On meta-analysis (Figure 2), there was no significant difference between stroke lateralization and fatigue; OR for left-side lateralization 0.88 (95% CI 0.64, 1.22) (*p* *=* 0.45). In view of the low-quality score of many studies (Supplementary sTable 8), we performed a sensitivity analysis excluding lower quality studies, defined as those with a quality score of below half of the maximum score from our meta-analyses (⩽3 for NOS Cross-sectional and ⩽4 for NOS Cohort study). Results remained nonsignificant (OR for left side: 0.86, 95% CI (0.57, 1.29), *p* = 0.46).

To assess whether the measure of fatigue used altered results, we performed a sensitivity analysis including only a single measure of fatigue (*n* = 7 studies^24,25,28,37,38,43,54^), and the result remained nonsignificant: OR for left side: 0.67, 95% CI [0.30, 1.47], *p* = 0.32.

#### Lesion location

Nineteen studies in 3036 subjects assessed whether there was an association between lesion location and fatigue.^1,7–9,19,27,29,31,33,38,43,46–53^ Lesion locations and study results are summarized in Table 2.

**Table 2.** Association of lesion location to PSF categorized by brain region.

	Lesion location	Studies	N	Results
Cortical	Cortical	Nine studies assessed cortical lesions.^1,8,33,38,44–48^	1748	One showed a small effect size for higher cognitive fatigue.^8^
	Insula	Two studies assessed insula lesions.^38,50^	65	Only one showed a difference in subjective anergia and underactivity and tiredness^50^
Subcortical	Subcortical	Four studies assessed subcortical lesions.^7,8,33,48^	467	One study showed an increased risk of physical and activity-related PSF with subcortical infarcts.^7^ Another study showed a medium effect size for cognitive and total fatigue and a small effect size for motor fatigue.^8^
	Basal Ganglia	Four studies assessed basal ganglia lesions.^1,44,46,47^	848	Only one study showed an association between increased risk of PSF with basal ganglia lesions.^47^
	Thalamus	Eight studies assessed thalamus lesions^29,43–48,51^	2042	One study showed an association between cerebellum, brainstem, and thalamus lesions with PSF at univariate, but not multivariate, level.^51^ Another study used VSLM to show right thalamus lesions were associated with fatigue at 6-month follow-up, but not baseline.^43^
	Internal Capsule	One study assessed internal capsule lesions.^47^	334	This study showed a higher number of internal capsule lesions in the fatigued group.^47^
	Caudate	One study assessed caudate lesions.^45^	500	This study showed an increased risk of PSF associated with caudate lesions.^45^
	Putamen	One study assessed putamen lesions.^45^	500	There were a higher number of putamen lesions in the fatigued group but no association with PSF on multivariate analyses.^45^
	Lacunar Infarcts	One study assessed lacunar infarcts.^19^	253	No association.
	Lenticulo-capsular area	One study assessed lenticulo-capsular lesions.^48^	220	No association.
	Globus Pallidus	One study assessed globus pallidus lesions.^45^	500	No association.
	Striatum-thalamus-frontal cortex	One study assessed striatum-thalamus-frontal cortex lesions.^38^	40	A trend for a higher number of these lesions in the fatigued group was observed.^38^
Infratentorial	Infratentorial	Five studies assessed infratentorial lesions.^1,7,9,44,46^	729	Two found an increased risk of PSF associated with infratentorial lesions.^7,9^
	Infratentorial v Supratentorial	Three studies assessed infratentorial lesions compared to supratentorial lesions.^27,31,48^	284	No association.
	Brainstem	Seven studies assessed brainstem lesions.^29,33,38,44,47,48,51^	1131	One found fewer brainstem lesions in the fatigued group.^47^ Another showed thalamus and/or brainstem lesions were associated with increased PSF risk.^29^ A third showed association between cerebellum, brainstem, and thalamus lesions with PSF at univariate, but not multivariate, level.^51^
	Cerebellum	Seven studies assessed cerebellum lesions.^33,38,44,45,47,48,51^	1530	One found fewer cerebellum lesions in the fatigued group.^47^ Another showed an association between cerebellum, brainstem, and thalamus lesions with PSF at univariate, but not multivariate, level.^51^
	Pons	Two studies assessed pons lesions.^45,48^	720	Only showed a reduced risk of PSF associated with pons lesions^45^
	Medulla	Two studies assessed medulla lesions.^45,48^	720	No association.
	Midbrain	Two studies assessed midbrain lesions.^45,48^	720	No association.
Cortical and Subcortical	Cortical and Subcortical	Two studies compared having both cortical and subcortical lesions.^8,9^	139	No association.
	Cortical-Subcortical	Two studies assessed cortical-subcortical lesions.^7,33^	216	No association.
Other	Infarct location	One study assessed infarct location.^49^	39	No association.

PSF: post-stroke fatigue; VSLM: voxel-symptom lesion-mapping.

*n* = 3036. Full study details and results are available in Supplementary sTable 3.

##### Cortical

Nine studies assessed cortical infarcts, with only one (*n* = 31) showing a weak association between cortical infarcts and cognitive fatigue.^8^ One study (*n* = 25) reported insular lesions to be associated with subjective anergia and tiredness after stroke,^50^ but a more recent study (*n* *=* 40) failed to replicate this finding.^38^ A further three studies testing overall differences between cortical, subcortical, and both cortical and subcortical lesions in PSF found no association.^7,9,33^

##### Subcortical

For the purposes of this review, we excluded brainstem and cerebellum from the category-defined subcortical structures and rather discussed them in the infratentorial section (see Table 2).

Four studies looked at associations with any subcortical lesions, of which two larger studies (*n* *=* 220,^48^ *n* *=* 109^33^) showed no association, while two smaller ones (*n* *=* 31,^8^ and *n* *=* 107^7^) reported an association (Table 2). Of the two positive studies, one reported association between subcortical lesions and higher motor fatigue, higher cognitive fatigue and total fatigue,^8^ while the second (*n* *=* 107) reported subcortical infarcts were associated with increased risk of physical PSF at 1 month (OR: 3.15, 95% CI (1.26, 7.86), *p* *=* 0.01) and at 3 months (OR: 2.56, 95% CI (1.07, 6.15), *p* *=* 0.04), and activity-related PSF at 1 month (OR: 2.96, 95% CI (1.17, 7.51), *p* *=* 0.02) and 6 months (OR: 2.71, 95% CI (1.12, 6.58), *p* *=* 0.03).^7^

Four studies assessed basal ganglia lesions.^1,44,46,47^ Three of these studies were conducted by the same research group; we contacted the corresponding author to enquire whether study populations were overlapping but did not receive a reply. Only one out of the three showed an association between acute basal ganglia infarcts and increased PSF risk (OR: 2.08, 95% CI (1.16, 3.75), *p* *=* 0.01) while controlling for sex, depression, instrumental activities of daily living and total number of acute infarcts.^47^

Eight studies assessed thalamic lesions,^29,43–48,51^ five studies, four by the same group, showed no association.^44–48^ One study showed thalamus and/or brainstem lesions were associated with PSF,^29^ while another found thalamus lesions to be associated with PSF in 230 participants.^51^ Another study used voxel-symptom lesion-mapping (VSLM) to show right thalamus lesions predicted PSF at follow-up (*n* = 324) when controlling for age, sex, lesion volume, hypertension, hypercholesterolemia, body mass index (BMI), diabetes, smoking, drinking, NIHSS score, stroke classification, depression and Lubben social score (OR: 2.67, 95% CI 1.46–4.88).^43^ However, this study showed no significant associations between any lesion location and acute fatigue (*n* = 361).^43^

Only one study (*n* *=* 334) assessed internal capsule lesions and found a higher number of lesions in the fatigued group.^47^ No other subcortical region investigated showed any association with PSF (Table 2).

##### Infratentorial

Infratentorial lesions were defined as: brainstem, cerebellum, pons, medulla, and midbrain (Table 2). Infratentorial lesions were investigated in eight studies. Six (*n* *=* 798) showed no association,^1,27,31,44,46,48^ while two found an association with increased PSF risk. One study in 108 participants showed infratentorial infarcts were associated with increased PSF risk at 6–8 weeks post-stroke (OR: 4.69, 95% CI 1.03-21.47), on multivariate analyses controlling for age, sex, anxiety, depressive symptoms, and disability.^9^ A second study in 107 subjects found an increased risk of global PSF at 3 months (OR: 2.91, 95% CI (1.24, 6.83), *p* *=* 0.01) and at 6 months (OR: 3.19, 95% CI (1.34, 7.58), *p* *=* 0.01) but not at 1 month.^7^

Of seven studies assessing brainstem lesions,^29,33,38,44,47,48,51^ one showed thalamus and/or brainstem lesions predicted general fatigue,^29^ while another (*n* = 230) showed brainstem lesions were associated with PSF on univariate analyses, but not when controlling for additional confounders.^51^ Two studies assessed specific brainstem regions (midbrain, pons, medulla),^45,48^ only one (*n* = 500) showed pons infarcts were associated with reduced PSF risk (OR: 0.47, 95% CI (0.26, 0.88), *p* *=* 0.02), after controlling for sex, depression, hyperlipidemia, Barthel Index score, and acute infarcts in the caudate and putamen.^45^ Seven studies assessed cerebellum lesions,^33,38,44,45,47,48,51^ one study (*n* = 334) showed fewer cerebellar infarcts in those with fatigue,^47^ while another (*n* = 230) showed cerebellar infarcts were associated with PSF at univariate level only.^51^

Four studies had either infratentorial group data or summary statistics suitable for meta-analysis.^7,9,27,46^ Two studies had data for multiple timepoints; we included only the earliest timepoint for consistency with other studies.^7,9^ On meta-analysis (sFigure 1), there was no significant association between infratentorial lesions and fatigue; OR: 1.83, 95% CI [0.63, 5.32] (*p* *=* 0.27). Excluding lower quality studies did not alter significance: OR: 1.78, 95% CI [0.54, 5.86], *p* = 0.34.

#### Lesion volume

Sixteen studies with 2755 participants tested the association between fatigue and lesion diameter or volume. ^1,7,20,21,31,32,35,43,44–49,52,53^ None identified any significant associations (see Supplementary sTable 4).

#### Brain atrophy

Four studies in 540 subjects examined brain atrophy measures in relation to the presence and severity of PSF^1,7,9,39^ (Supplementary sTable 5). All examined associations with global brain volumes, including some subcortical and cortical volumes, and none reported association with PSF. One study (*n* = 107) examined specific brain regions and reported no significant associations with PSF.^39^

#### Number of infarcts

Four studies, all from the same author, assessed whether the number of acute infarcts was associated with PSF^44–47^ (*n* *=* 1130) (Supplementary sTable 6). An initial study showed more acute infarcts in those with PSF,^47^ but three subsequent studies failed to confirm this finding.^44–46^

Four studies in 922 subjects assessed the relationship between the number of old infarcts and the presence of PSF (Supplementary sTable 6). All studies showed no significant relationship.^1,39,44,45^

#### Cerebral microbleeds

Only one study assessed cerebral microbleeds (CMBs) and PSF in 199 subjects.^46^ Deep CMBs (basal ganglia, external capsule, internal capsule, and thalamus) were associated with fatigue on univariate analysis (*p* *=* 0.038) and on multivariate analyses in the same population (OR: 2.68, 95% CI: (1.20, 6.00), *p* *=* 0.02) controlling for age, depression, and total number of CMBs.^46^

#### WMHs

Seven studies in 1336 subjects assessed the relationship between WMH and PSF^1,7,9,39,44–46^ (Supplementary sTable 7). All used visual rating scales to assess WMH severity (Fazekas scale in 5, ARWMC scale in 1, and one bespoke grading scale). Six showed no association between WMH severity and PSF.^1,9,39,44–46^ One reported an association with a Fazekas score of 1 and mental fatigue at 3 (OR: 1.54, 95% CI (1.05, 2.21), *p* *=* 0.03) and 6 months (OR: 1.79, 95% CI (1.20, 2.65), *p* *=* 0.04) but not at 1 month.^7^

Three studies had group data or summary statistics available for meta-analysis.^7,9,45^ On meta-analysis (sFigure 2), there was no association between WMH and fatigue; OR: 1.21, 95% CI [0.84, 1.75], (*p* *=* 0.29).

#### Diffusion tensor imaging (DTI) of white matter ultrastructure

DTI is a sensitive marker of white matter ultrastructural damage in cerebrovascular disease and has been correlated with symptoms such as cognition^58,59^ and apathy^60^ in stroke. At the time of the search strategy, we were unable to find any papers assessing DTI parameters and PSF.

#### Brain connectivity and network analysis

It has been suggested that cognitive and psychological symptoms arise from damage to anatomically distributed brain networks caused by focal lesions rather than specific lesion locations themselves.^59^ Network disruption is important in mediating the effect of cerebral small-vessel disease on both cognition^58,61^ and apathy.^60^ Networks can be assessed structurally using diffusion-weighted imaging and tractography and functionally using functional MRI (fMRI).

Two studies investigated structural tractography-based networks and their relationship to PSF.^35,52^ The first study constructed individual-level whole-brain disconnectivity probability maps based on lesion maps from 84 stroke patients using normative data from healthy controls.^35^ Nonparametric permutation-based inference was used to conduct voxel-wise analyses on disconnectome maps and estimate regional disconnectivity.^35^ There was no association between PSF and any disconnectome measures after accounting for multiple comparisons and controlling for depression.^35^ A second smaller study used diffusion MRI data from 420 healthy controls to map structural disconnection caused by lesions to the white matter in 12 post-stroke participants.^52^ When all lesions were lateralized to the same hemisphere, voxel-wise correlations showed higher fatigue correlated with greater structural disconnection in an ipsilesional cluster (rostral middle frontal cortex and superior frontal cortex) and a contralesional cluster containing six frontal cortex regions (rostral middle frontal cortex, caudal middle frontal cortex, superior frontal cortex, inferior frontal cortex (pars triangularis and pars opercularis), and caudal anterior cingulate cortex).^52^ When lesions were not lateralized to the same hemisphere, no significant associations were observed.^52^

Three studies assessed resting-state brain activity markers of PSF using fMRI.^21,38,52^ In 63 stroke subjects, PSF was associated with posterior hypoactivity and prefrontal hyperactivity, as assessed by the amplitude of low-frequency fluctuations (ALFF), which was suggested to reflect dysfunction within large-scale brain systems such as fronto-striatal-thalamic and frontal-occipital networks.^21^ A second study in 16 post-stroke patients reported fractional ALFF was lower in several brain areas in PSF, including the right frontal, right inner orbital frontal, right orbital inferior frontal, right triangular frontal inferior, right anterior and lateral cingulate, and right medial frontal gyri.^38^ A third small study (*n* = 12) used fMRI to show that reduced network functional connectivity in the ipsilesional rostral middle frontal cortex was associated with higher fatigue severity.^52^

Two studies assessed the effect of modafinil on fMRI functional connectivity (FC) in PSF,^55,56^ using data from the double-blind MIDAS-II trial (*n* *=* 36), which showed modafinil 200 mg daily reduced PSF compared to placebo.^62^ In a subgroup of 28 who had fMRI, modafinil treatment was associated with increased resting-state functional connectivity (rsFC) in the right hippocampus compared to placebo, and lower rsFC in the left frontoparietal, somatosensory, and mesolimbic network.^55^ In a second subgroup study (*n* = 23), FC between the dorsolateral prefrontal cortex and the caudate nucleus was a significant predictor of reduced PSF after modafinil use.^56^ The authors concluded that fronto-striato-thalamic FC predicted modafinil response for PSF.^56^

Two studies tested whether disruption of brain asymmetry was associated with PSF.^54,57^ The first (*n* = 18) assessed inter-hemispheric inhibition balance (IIB) using resting-state fMRI (rs-fMRI) and paired-pulse transcranial magnetic stimulation (TMS).^57^ rs-fMRI results showed that fatigue score was predicted by IIB in the primary motor cortex (M1) but not in the insula, caudate, or thalamus.^57^ Paired-pulse TMS in 41 participants also supported an association between IIB in the M1 and PSF.^57^ A second study used paired-pulse TMS to show a significant positive correlation between fatigue severity and intracortical facilitation (ICF) asymmetry in a subgroup of participants with fatigue (*n* = 14) but no association in the whole cohort (*n* = 20).^54^

### Risk of Bias Assessment

Using the NOS, we identified a moderate level of bias; with a maximum score of 8 or 9 (cross-sectional and cohort studies, respectively) mean (SD) quality score was 4.57(1.14) (Supplementary sTable 1 for individual study bias estimates).

## Discussion

In this systematic review and meta-analysis, carried out using robust methodology according to PRISMA guidelines and including a total of 46 papers (n *=* 6543 subjects), we found no robust relationship between PSF and structural imaging features. Although a minority of papers reported associations, these were inconsistent, and most studies reported no association. Considering the totality of the data, we found no clear evidence of any association between lesion location or laterality, number of infarcts, WMHs, brain atrophy, or CMBs and PSF. There was sufficient data to perform a meta-analysis for lesion lateralization, WMH, and infratentorial lesion location, and these were all negative, supporting a lack of any association.

It has been suggested that a number of the cognitive and psychological consequences of stroke may be mediated by damage to distributed brain networks.^58,59,61^ We found limited studies investigating associations between network integrity and PSF. Structural network integrity relies on the reconstruction of white matter connections using tractography analysis of DTI. One large study utilizing this technique found no association.^35^ Of note, the same technique applied to the same dataset was able to show an association between network integrity and post-stroke cognitive impairment, suggesting the technique does have the power to detect associations between symptoms and network dysfunction.^59^ However, a second small study found structural disconnection in several ipsilesional and contralesional frontal brain regions, as well as reduced FC in the ipsilesional rostral middle frontal cortex, associated with PSF.^52^

Functional network measures rely on the BOLD MRI signal and temporal correlations between activation in different brain regions. In small or moderate-sized cohorts, these did suggest some potential associations with PSF.^21,38,52,55,56^ It has been hypothesized that fatigue in neurological disorders may relate to alterations in limbic input and motor functions in the basal ganglia, therefore, affecting activity in the fronto-striato-thalamic system.^63^ Consistent with this, one fMRI study identified posterior hypoactivity and prefrontal hyperactivity, which was interpreted to reflect dysfunction within fronto-striatal-thalamic and frontal–occipital networks.^21^ The MIDAS-II drug trial identified reduced connectivity of the fronto-striatal-thalamus predicted better response to modafinil.^56^ With both structural and functional studies implicating frontal regions,^21,52,56^ future work should further investigate the role of frontal networks in PSF.

Altered perceived effort may also underlie fatigue; it has been suggested that reduced ability to attenuate irrelevant sensory stimuli drives increased perceived effort and causes feelings of fatigue.^64^ One functional network study showed those whose fatigue responded to modafinil treatment had significant decreases in rsFC in the somatosensory cortex, inferior parietal lobule, and temporal pole.^55^ This may indicate changes within brain areas associated with sensory processing. Specifically, increased activity in these regions at rest, prior to modafinil use and fatigue improvement, could be reflective of overactivity in sensory processing leading to poorer attenuation and, as a result, fatigue. Furthermore, Ondobaka et al.^57^ used rs-fMRI to show that IIB in the M1 was associated with, and could predict, fatigue score. The authors suggest that altered brain asymmetry in the M1 may cause sympathetic central nervous system dominance, leading to higher arousal, poorer sensory attenuation, and, thus, fatigue.^57,65^

Taken together, our review suggests anatomical characteristics of the stroke lesion do not relate to PSF. In particular, lesion size and laterality are not important in the pathogenesis of PSF, suggesting clinical care should focus on screening and management of known risk factors, such as inactivity^66^ and mood,^67^ in order to manage PSF.

Whether there is a lack of any relation, or whether the analysis techniques applied have been too simplistic remains uncertain. Taken together, lesion location studies were largely inconclusive, with no consistent associations with any particular region; however, one large VSLM study implicated the right thalamus in PSF at 6 months post-stroke but not at baseline.^43^ Future research should utilize such advanced imaging techniques to further probe the relationship between fatigue and lesion location. It is also possible a more network-based approach is required to identify relationships, and early studies have implicated the frontal,^38,52^ fronto-striato-thalamic,^21,56^ and sensory processing networks,^55,57^ but further studies are required to replicate these associations.

It is also possible that perhaps the fatigue measures may have been too simplistic to detect associations between neuroimaging parameters and fatigue. Most studies we identified treated fatigue as a single construct, but this may not be the case. Emerging evidence suggests the cause of PSF is multidimensional.^6^ Studies have investigated demographic factors, neurological/physical deficits, medical comorbidities, smoking, medications, sleep disturbances, pain, pre-stroke fatigue, depression and anxiety, and cognitive impairment as potential risk factors.^6^ It is, therefore, possible that the same subjective experience of fatigue could result from a number of different mechanisms. The varied response to the modafinil treatment in the MIDAS-II trial further corroborates the heterogenous nature of fatigue.^55,56^

Future research could investigate the inflammation hypothesis: inflammatory response following stroke upregulates proinflammatory cytokines causing sickness behaviors, further leading to feelings of fatigue.^68^ Early studies have shown associations between fatigue after stroke and inflammatory markers,^6,31,68^ suggesting a viable avenue for future research.

One limitation of this review is the moderate risk of bias displayed by studies; this is likely to be partially explained by the nature of included studies. Many studies were not designed to specifically investigate fatigue and neuroimaging, rather a number examined these as secondary or subanalyses within existing research/research questions (28.3%) (Supplementary sTable 9), highlighting the infancy of this field and the need for more robust research assessing the pathogenesis of PSF. Some studies included were primary, looking for associations between neuroimaging parameters and fatigue; in others, it was a secondary question. However, sensitivity analysis removing secondary analyses from the meta-analyses did not significantly alter results for either lesion lateralization or infratentorial location (results not shown). Additional sensitivity analysis excluding lower-quality studies also did not alter the results of the meta-analyses.

The meta-analyses combined studies regardless of fatigue outcome measure used. As noted above, fatigue is a multidimensional construct, and different outcome measures may measure different, and perhaps not overlapping, domains.^66^ For the lesion lateralization meta-analysis, there were sufficient studies to perform a sensitivity analysis using only a single measure of fatigue, and this did not alter the results significantly.

Of note, many studies did not control for confounders in the current review; in fact, most carried out only univariate analyses. Some studies did control for key confounders such as age,^9,21,35,43–45^ sex^9,21,43–45^ and depression;^1,9,21,29,35,43–45^ however, the majority did not. Age and sex have both been previously associated with fatigue,^6^ while depression is a known major confounder in the assessment of fatigue.^6^ It is key that future research should consider and control for these factors, to accurately identify potential correlates of fatigue.

Finally, many studies considered both ischemic and hemorrhagic stroke, it is important to note that differences in stroke pathophysiology could also mean differing pathomechanisms of fatigue between types of stroke. Future research should aim to assess differences in fatigue across types of stroke.

In conclusion, we found little evidence for any association of neuroimaging features with PSF, although for some imaging variables, there was limited data available. Several studies implicated altered functional brain networks in PSF, but more work is required to confirm and fully understand the relevance and clinical implications of such alterations.

## Supplemental Material

sj-docx-1-wso-10.1177_17474930231192214 – Supplemental material for Neuroimaging correlates of post-stroke fatigue: A systematic review and meta-analysis Click here for additional data file. Supplemental material, sj-docx-1-wso-10.1177_17474930231192214 for Neuroimaging correlates of post-stroke fatigue: A systematic review and meta-analysis by Amy A Jolly, Adriana Zainurin, Gillian Mead and Hugh S Markus in International Journal of Stroke
