# Supplementary material for: Neuroimaging correlates of post-stroke fatigue: A systematic review and meta-analysis
Source: Int J Stroke. 2023 Aug 12;18(9):1051–62. doi: 10.1177/17474930231192214 (PMC10614177; doi:10.1177/17474930231192214)
Supplement: sj-docx-1-wso-10.1177_17474930231192214 – Supplemental material for Neuroimaging correlates of post-stroke fatigue: A systematic review and meta-analysis [file sj-docx-1-wso-10.1177_17474930231192214.docx]

**Supplementary Material V2**

Appendix 1: Search Strategy

Appendix 2: Additional References for sFigure 2

sFigure 1. Forest plot showing the association between infratentorial lesions and prevalence of PSF.

sFigure 2. Forest plot showing the association between WMHs and prevalence of PSF.

sTable 1: Table to show risk of bias assessments as rated by NOS scale.

sTable 2: Characteristics of studies included in the systematic review.

sTable 3: Table to show characteristics and findings of studies assessing lesion location.

sTable 4: Table to show characteristics and findings of studies assessing lesion volume or lesion size.

sTable 5: Table to show characteristics and findings of studies assessing brain atrophy.

sTable 6: Table to show characteristics and findings of studies assessing infarct number (both acute and old).

sTable 7: Table to show characteristics and findings of studies assessing white matter hyperintensities.

sTable 8. Table to show characteristics of studies included in each meta-analysis.

sTable 9. Table to show study aim and whether studies were primary or secondary analyses.

**Appendix 1**

To be included in record screening, records had to meet searches 1, 2 and 3 (AKA search 4).

Search terms were:

1. **(fatigue OR ("fatigue syndrome" OR "chronic fatigue" OR "asthenia" OR "poststroke fatigue" OR "post-stroke fatigue" OR "post stroke fatigue" OR "subjective fatigue" OR "central fatigue" OR "mental fatigue" OR "muscle fatigue" OR "tired" OR "tiredness" OR letharg* OR "malaise" OR "listlessness" OR "lassitude" OR exhaust* OR wear* OR astheni* OR neuroastheni* OR fatigue*))**
2. **("stroke" OR "cerebrovascular disorders" OR "cerebrovascular accident" OR "cerebrovascular event" OR cerebrovasc* OR cva OR apoplex* OR "acute stroke" OR "brain attack" OR "brain insult" OR "cerebral stroke" OR ischaemic OR ischemic OR "ischaemic stroke" OR "ischemic stroke" OR "brain thrombo*" OR "brain haemorrhage" OR "brain hemorrhage" OR infarct*)**
3. **(MRI OR "MR imaging" OR "MR" OR "MRI scan" OR "Magnetic Resonance Imaging" OR neuroradiography OR "brain scan" OR "neuroimaging correlate*" OR neuroimaging OR "functional neuroimaging" OR "CT" OR tomography OR "computed tomography" OR "emission computed tomography" OR "white matter" OR "white matter lesion*" OR "white matter hyperintens*" OR Leucoaraiosis OR Leukoaraiosis OR ""lesion load" OR "lesion volume" OR "brain volume" OR "brain atrophy" OR "cerebral atrophy" OR "lesion" OR "lesion location" OR "lesion site" OR ""lesion side" OR lateralisation OR lateralization OR hemisphere OR cortex OR "cerebral hemisphere" OR "brain area" OR "diffusion tensor imaging" OR DTI OR "positron emission tomography" OR PET OR inflamm* OR connect* OR disconnect*)**
4. 1+2+3

**Appendix 2**

Additional References for sFigure 2

69. Hozo SP, Djulbegovic B, Hozo I. Estimating the mean and variance from the median, range, and the size of a sample. *BMC Med Res Methodol* 2005; 5: 13.

70. Deeks JJ, Higgins JPT, Altman DG (editors). Chapter 10: Analysing data and undertaking meta-analyses. In: Higgins JPT, Thomas J, Chandler J, et al. (eds) *Cochrane Handbook for Systematic Reviews of Interventions version 6.3 (updated February 2022)*. Cochrane, 2022.

***sFigure 1*. Forest plot showing the association between infratentorial lesions and prevalence of PSF.**

*
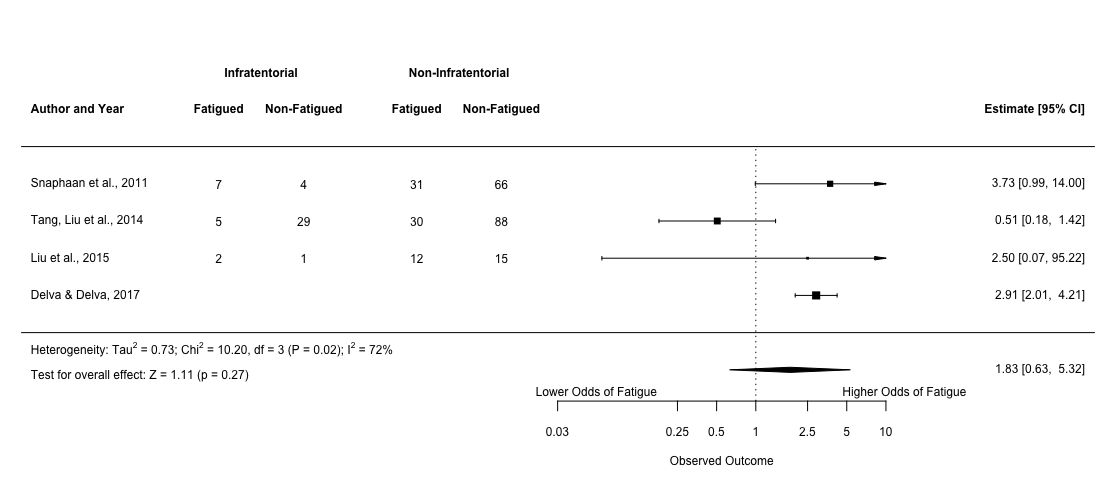
*

*sFigure 1 Note*. Liu et al. 2015^27^ looked at low-moderate vs. severe fatigue. Snaphaan et al. 2011^9^ group data calculated using percentages.

***sFigure 2.*** **Forest plot showing the association between WMHs and prevalence of PSF.**

*
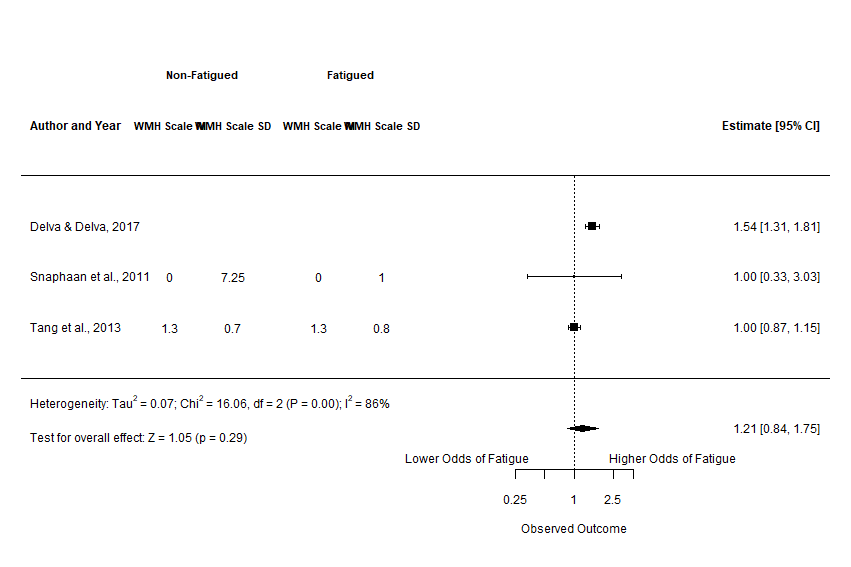
*

*sFigure 2 Note.* Snaphaan et al. 2011^9^ group data converted from Mdn(range) to M(SD) using Hozo et al. 2005.^69^ Snaphaan et al. 2011^9^ and Tang et al. 2013^47^ mean-standard differences re-expressed as ORs as detailed in Cochrane Handbook (Section 10.6).^70^

**sTable 1: Table to show risk of bias assessments as rated by the Newcastle-Ottawa Scale (NOS).**

| sTable 1. Table to show risk of bias assessments as rated by NOS. | | | | | |
| --- | --- | --- | --- | --- | --- |
| **Study** | **NOS Scale used** | **Selection** | **Comparability** | **Outcome** | **Total** |
| Appelros, 2006 | NOS Cross-sectional | 3 | 0 | 2 | 5 |
| Becker et al., 2015 | NOS Cross-sectional | 1 | 0 | 2 | 3 |
| Chen et al., 2015 | NOS Cross-sectional | 2 | 0 | 2 | 4 |
| Chen & Marsh, 2018 | NOS Cross-sectional | 3 | 0 | 2 | 5 |
| Choi-Kwon et al., 2005 | NOS Cross-sectional | 2 | 0 | 2 | 4 |
| Cotter et al., 2022 | NOS Cross-sectional | 2 | 1 | 2 | 5 |
| De Doncker et al., 2021 | NOS Cross-sectional | 1 | 0 | 2 | 3 |
| Delva & Delva, 2017 | NOS Cross-sectional | 2 | 0 | 2 | 4 |
| Delva et al., 2017 | NOS Cross-sectional | 2 | 0 | 2 | 4 |
| Duncan et al., 2015 | NOS Cross-sectional | 4 | 0 | 2 | 6 |
| Hubacher et al., 2012 | NOS Cross-sectional | 2 | 0 | 2 | 4 |
| Jaracz et al., 2007 | NOS Cross-sectional | 2 | 0 | 2 | 4 |
| Khan & Delargy, 2017 | NOS Cross-sectional | 2 | 0 | 1 | 3 |
| Kindred et al., 2023 | NOS Cross-sectional | 2 | 0 | 2 | 4 |
| Kjeverud et al., 2020 | NOS Cohort | 3 | 0 | 3 | 6 |
| Kutlubaev et al., 2013 | NOS Cross-sectional | 3 | 1 | 2 | 6 |
| Lamb et al., 2013 | NOS Cross-sectional | 2 | 0 | 2 | 4 |
| Liu et al., 2015 | NOS Cross-sectional | 1 | 0 | 2 | 3 |
| Lynch et al., 2007 | NOS Cross-sectional | 3 | 0 | 2 | 5 |
| Manes, 1999 | NOS Cross-sectional | 1 | 0 | 2 | 3 |
| Marsh et al., 2018 | NOS Cross-sectional | 2 | 0 | 2 | 4 |
| Miller et al., 2013 | NOS Cross-sectional | 1 | 0 | 2 | 3 |
| Mutai et al., 2017 | NOS Cross-sectional | 2 | 1 | 2 | 5 |
| Naess et al., 2005 | NOS Cross-sectional | 1 | 0 | 2 | 3 |
| Ondobaka et al., 2022 | NOS Cross-sectional | 2 | 2 | 2 | 6 |
| Ormstad et al., 2011 | NOS Cross-sectional | 2 | 0 | 2 | 4 |
| Pihlaja et al., 2014 | NOS Cross-sectional | 2 | 0 | 2 | 4 |
| Ponchel et al., 2016 | NOS Cross-sectional | 2 | 0 | 2 | 4 |
| Radman et al., 2012 | NOS Cross-sectional | 3 | 0 | 3 | 6 |
| Schaechter et al., 2023 | NOS Cross-sectional | 1 | 0 | 2 | 3 |
| Schepers et al., 2006 | NOS Cross-sectional | 2 | 0 | 2 | 4 |
| Snaphaan et al., 2011 | NOS Cross-sectional | 3 | 1 | 2 | 6 |
| Stein et al., 1996 | NOS Cross-sectional | 1 | 0 | 2 | 3 |
| Tang et al., 2010 | NOS Cross-sectional | 2 | 1 | 2 | 5 |
| Tang et al., 2013 | NOS Cross-sectional | 3 | 1 | 2 | 6 |
| Tang, Liu et al., 2014 | NOS Cross-sectional | 2 | 1 | 2 | 5 |
| Tang, Chen et al., 2014 | NOS Cohort | 3 | 1 | 2 | 6 |
| Tynterova et al., 2023 | NOS Cross-sectional | 2 | 0 | 2 | 4 |
| Ulrichsen et al., 2021 | NOS Cross-sectional | 2 | 2 | 2 | 6 |
| Van Eijsden et al., 2012 | NOS Cohort | 3 | 0 | 3 | 6 |
| Visser, Goodin et al., 2019 | NOS Cross-sectional | 3 | 0 | 2 | 5 |
| Visser, Maréchal et al., 2019 | NOS Cross-sectional | 2 | 0 | 2 | 4 |
| Wang et al., 2014 | NOS Cross-sectional | 4 | 0 | 2 | 6 |
| Wang et al., 2022 | NOS Cross-sectional | 3 | 2 | 2 | 8 |
| Zhang et al., 2021 | NOS Cross-sectional | 1 | 1 | 2 | 4 |
| Zhang et al., 2023 | NOS Cross-sectional | 3 | 1 | 2 | 6 |
| *Note.* Higher scores indicate lower risk of bias/higher quality of study.  NOS Cross Sectional scoring: Selection total possible score = 4, Comparability total possible score= 2, Outcome total possible score = 2. Overall maximum score = 8.  NOS Cohort total: Selection total possible score = 4, Comparability total possible score= 2, Outcome total possible score = 3. Overall maximum score = 9. | | | | | |

**sTable 2: Characteristics of studies included in the systematic review.**

| sTable 2. Characteristics of studies included in the systematic review. | | | | | | | | |
| --- | --- | --- | --- | --- | --- | --- | --- | --- |
| **Study** | **Sample Size** | **Population** | **Age (M (SD))** | **Gender (% male)** | **Time since Stroke** | **Primary Fatigue Measure** | **How is Fatigue Categorised?** | **MRI variables investigated** |
| Appelros, 2006 | 253 | First ever ischaemic stroke patients. | 74.5 (not reported) | 49% | 12M | Self-report | Yes or no | Lesion Lateralisation, Lesion Location |
| Becker et al., 2015 | 39 | Ischaemic stroke patients, 18+.  No history of brain tumour, anaemia, HIV, hepatitis B or C or those taking immunomodulatory medication. | Overall not reported | 58.97% | 1M, 3M, 6M and 12M | FAS | Scores of: 10-21 (no fatigue), 22-34 (fatigue) and 35-50 (extreme fatigue) | Lesion Location, Lesion Volume |
| Chen et al., 2015 | 218 | Acute or recurrent ischaemic stroke, aged 40-80.  No dementia or cognitive impairment, severe medical conditions, NIHSS ≥ 15, or recurrent stroke (<3 months). | 61.2 (11.4) | 73.4% | <3M | FSS | Average score of ≥ 4.0 | Brain Atrophy, Lesion Location, Lesion Volume, Number of Infarcts, White Matter Hyperintensities |
| Chen & Marsh, 2018 | 203 | Ischaemic and haemorrhagic stroke patients within 6 months. | 65.2 (15.7) | 50% | Mean (SD) days:  48.4 (24.2) | FACIT | <41 = any fatigue, <31 = severe fatigue. | Lesion Lateralisation, Lesion Volume |
| Choi-Kwon et al., 2005 | 220 | Outpatients with between 40-80, <3 months post-stroke.  No previous history of stroke or bilateral/multiple lesions on MRI, no miscellaneous aetiologies, no MRS ≥4, TIA, aphasia, dementia, depression, cognition dysfunction or living alone. | 60 (not reported) | 73.7% | > 3 months (mean= 15M) | VAS | Mild (1–3), Moderate (4–6), Severe (7–10) | Lesion Location, Lesion Volume |
| Cotter et al., 2022 | 63 | Ischaemic stroke patients. | 67.51 (13.39) | 65.08% | 3M, 12M and 3 years | PHQ-9 (item 4) | >0 | Lesion Lateralisation, Lesion Volume, Network Measures |
| De Doncker et al., 2021 | 73 | First ischaemic or haemorrhagic stroke at least 3 months prior, with sufficient grip strength.  No other neurological disorder, depression, anti-depressants/ centrally acting medicines, sensory impairment, or contraindications to TMS. | 61.55 (12.11) | 67.1% | >3 months | FSS-7 | Average score. Higher scores = higher fatigue | Lesion Lateralisation |
| Delva & Delva 2017 | 107 | Acute ischaemic stroke patients with MRI.  No major medical illness that could cause fatigue, alcohol abuse, problems with consciousness, depression, anxiety, cognitive impairment, impaired speech, language or writing that prevented participation and no MRS ≥4. | 64.6 (8) | 47.7% | 1M, 3M and 6M | MFI-20 | A score 12+ (per subscale) | Brain Atrophy, Lesion Location, Lesion Volume, White Matter Hyperintensities |
| **Study** | **Sample Size** | **Population** | **Age (M (SD))** | **Gender (% male)** | **Time since Stroke** | **Primary Fatigue Measure** | **How is Fatigue Categorised?** | **MRI variables investigated** |
| Delva et al., 2017† | 285 (234 with stroke) | Stroke patients.  No major medical illness that could cause fatigue, alcohol abuse, problems with consciousness, depression, anxiety, cognitive impairment, impaired speech, language or writing that prevented participation and no MRS ≥4. | 63.1 (8.7) | 48.1% | Acute, 1M and 3M | MFI-20 | A score 12+ (per subscale) | Lesion Lateralisation |
| Duncan et al., 2015 | 136 | Ischaemic or haemorrhagic stroke within past month in south Edinburgh.  No SAH, medical instability, dysphasia or impaired cognition preventing informed consent or questionnaire completion. | 71.8 (62.6- 79.2)* | 64.7% | 1M, 6M and 12M | FAS | Continuous score. Higher scores = higher fatigue. | Lesion Lateralisation |
| Hubacher et al., 2012 | 31 | Stroke patients between 35-76.  No other neurological or psychiatric disorder, aphasia or incomplete questionnaire data. | 59.29 (10.3) | 80.65% | Mean (days): 50.65 | FSMC | Scores for Cognitive and Motor subscales and Sum of Scores. | Lesion Location |
| Jaracz et al., 2007 | 50 | First ever stroke patients.  No serious cognitive or speech disturbances, concomitant diseases, or pre-stroke restrictions in activities of daily living. | 55 (7.73) | 66% | 3M | FIS (Polish Version) | Continuous score. Higher scores = higher fatigue. | Lesion Lateralisation |
| Khan & Delargy, 2017 | 30 | Stroke inpatients, >18, admitted for stroke in last 6 months. | Overall not reported | 70% | <6 months | FSS | Not reported, but binary (Fatigued/ Non-fatigued) | Lesion Lateralisation |
| Kindred et al., 2023 | 27 | Participants aged 35-80, >6-months post-stroke, able to move 10m without assistance and measurable TMS response.  No comorbid neurological disease, history of major head trauma, contraindication to MRI or inability to follow instruction. | 63.55 (8.43) | 65% | >6 months | FSS | Total score ≥36 | Lesion Lateralisation |
| Kjeverud et al., 2020 | 115 | First ever stroke (ICD classification).  No cognitive impairment limiting participation or unconsciousness. | 68.3 (13.3) | 59% | Acute, 6M, 12M and 18M | FSS | Low, Moderate and High fatigue groups | Lesion Lateralisation |
| **Study** | **Sample Size** | **Population** | **Age (M (SD))** | **Gender (% male)** | **Time since Stroke** | **Primary Fatigue Measure** | **How is Fatigue Categorised?** | **MRI variables investigated** |
| Kutlubaev et al., 2013 | 107 | Ischaemic or haemorrhagic stroke patients.  No SAH, severe dysphasia or cognitive impairment. Those with no CT were excluded, | 70.5 (62-77)* | 62% | 1M | FAS | Continuous score. Higher scores = higher fatigue. | Brain Atrophy, Lesion Lateralisation, Number of Infarcts, White Matter Hyperintensities |
| Lamb et al., 2013 | 25 | First ever ischaemic stroke patients between 50-80.  No history of psychiatric illness, neurological disease, drug/alcohol abuse, dementia, moderate to severe aphasia, or insufficient English to allow neuropsychological testing. | 67 (10) | 64% | Mean (SD) (months): 6.64 (1.32) | MFI | ≥12 on General Fatigue Scale | Lesion Lateralisation |
| Liu et al., 2015 | 30 | Clinically and radiologically confirmed CVA >3 months prior to study and aged 18-90.  No significant disability pre-stroke, reduced alertness, language reception or attention impacting participation, excessive pain, severe disease, terminal diagnosis (<1 year), drug or alcohol abuse within 3 years, pregnancy or participation in other research studies. | 55.6 (14.5) | 46.67% | >3 months | FSMC | Low-moderate fatigue/ Severe fatigue (not specified how) | Lesion Lateralisation, Lesion Location |
| Lynch et al., 2007 | 55 | New stroke patients.  No medical instability due to other conditions, dysphasia or confusion preventing participation. | 73 (66-81)* | 56% | Mdn (IQR) (days): 23 (93-217) | Self-report in line with case-definition | Meet case definition/ do not meet case definition | Lesion Lateralisation |
| Manes, 1999 | 25 | Single lesion on CT and/or MRI showing ischaemic infarct in insula or other cortical area.  No frontal lobe lesions, severe deficits preventing participation or significantly reduced consciousness. | Not reported | 76% | 4-8 weeks | Modified Present State Exam | Subjective anergia and underactivity /  Tiredness | Lesion Location |
| Marsh et al., 2018 | 151 | Acute stroke on imaging seen at clinic 3-months post-stroke.  No NIHSS ≥4. | 67.1 (13.8) | 49% | Mean (SD) days:  83.6 (190) | FACIT | Total score of <30 = severe fatigue | Lesion Volume |
| Miller et al., 2013 | 77 | Participants with chronic stroke (>6 months) aged between 50-85 with residual deficit, who had been referred to OT or PT at time of stroke and completed inpatient rehabilitation.  No history of psychiatric history with inpatient stay or impaired verbal communication. | 64.1 (not reported) | 75.3% | >6 months | FSS | Average score of ≥ 4.0 | Lesion Lateralisation |
| **Study** | **Sample Size** | **Population** | **Age (M (SD))** | **Gender (% male)** | **Time since Stroke** | **Primary Fatigue Measure** | **How is Fatigue Categorised?** | **MRI variables investigated** |
| Mutai et al., 2017 | 101 | Ischaemic or haemorrhagic stroke patients.  Severe confusion, aphasia or motor complications were not excluded. | 74.4 (11.6) | 66.3% | <2 weeks | MFI | Continuous scores within 5 subscales: General/ physical/ reduced activity/ reduced motivation/ mental fatigue. Higher scores = higher fatigue. | Lesion Lateralisation, Lesion Location |
| Naess et al., 2005 | 192 | Patients given diagnosis of stroke at hospital contacted retrospectively.  No SAH, sinus venous thrombosis or severe head trauma. | 47.8 (Not reported) | 57.3% | Mean (years): 6 | FSS | Average score of ≥ 4.0 | Lesion Lateralisation |
| Ondobaka et al., 2022 | 59 | First time ischaemic or haemorrhagic stroke patients aged 18+, >3 months from stroke.  No centrally acting medicines, contraindications to MRI or TMS, depression, or poor limb function. | Overall not reported | 81.4% | >3 months | FSS-7 | Average score. Higher scores = higher fatigue. | Network Measures |
| Ormstad et al., 2011 | 45 (34 with imaging) | First ever stroke patients aged 18+.  No cognitive impairment, uncertain acute ischaemic stroke diagnosis or uncertain symptom onset, cancer or those receiving thrombolysis. Those with no samples taken were also excluded. | 67.7 (11.8) | 60% | Acute, 6M, 12, 18M | FSS | Average score of ≥ 4.0 | Lesion Lateralisation (n = 41), Lesion Location (n = 34), Lesion Volume (n = 34) |
| Pihlaja et al., 2014 | 133 | First ever supratentorial ischaemic stroke patients with Finnish as native language.  No history of neurological or severe psychiatric history. | 54.6 (9.5) | 64.7% | Mean (SD) days 84.7 (23.9) | Profile of Mood States-F and Profile of Moods States-V | Cut off as the 90th percentile of combined subscales of control group. | Lesion Lateralisation, Lesion Volume |
| Ponchel et al., 2016 | 153 | Ischaemic or haemorrhagic stroke (confirmed on MRI), 18+.  No pre-stroke dementia, those under legal guardianship, non-French speaking, contraindication for MRI, pure-meningeal, malformed, traumatic, or intraventricular haemorrhages, or neurological deficits or aphasia severe enough to prevent taking part. | 63.6 (12.8) | 60.8% | 6M | Chalder Fatigue Scale | 4+ | Lesion Lateralisation |
| **Study** | **Sample Size** | **Population** | **Age (M (SD))** | **Gender (% male)** | **Time since Stroke** | **Primary Fatigue Measure** | **How is Fatigue Categorised?** | **MRI variables investigated** |
| Radman et al., 2012 | 109 | Minor acute ischaemic or haemorrhagic stroke patients (<7 days) with regular involvement in working activities prior to stroke.  No Rankin scores above <1 at 6 months, no one above $\geq$70 years old, impairment in daily life pre-stroke, recurrent stroke, TIA, SAH, psychiatric history, sleep disorders or comorbid diseases that could affect fatigue assessment. | 51.1 (13.8) | 66% | <7 days, 6M and 12M | FAI | Total FAI severity score | Lesion Lateralisation, Lesion Location |
| Schaechter et al., 2022 | 12 | Stroke patients with unilateral ischaemic stroke in the middle cerebral artery 1-3 years prior.  No significant pre-stroke fatigue, significant pre-stroke disability, significant cognitive deficit, NIHSS 1b and 1c ≥1 or 9 ≥2, contraindication to MRI or neurological disorder other than chronic stroke. No major kidney or liver disease, active infection, recent use of drugs with high TSPO binding or systemic immunomodulators, or low-affinity TSPO binding based on genotype. | 56/08 (11.91) | 66.67% | 1-3 years post-stroke. (M(SD) = 1.72 (0.43) years). | General Fatigue subscale of MFI | ≥12 and continuous score (higher scores= higher fatigue) | Lesion Lateralisation, Lesion Volume, Network Measures |
| Schepers et al., 2006 | 167 | First ever stroke patients admitted for inpatient rehabilitation with unilateral supratentorial lesion.  No disabling comorbidity or aphasia. Non-Dutch speakers were also excluded. | 56.4 (11.4) | 58.7% | Admission, 6M, 12M | FSS | Average score of ≥ 4.0 | Lesion Lateralisation |
| Snaphaan et al., 2011 | 108 | Acute symptomatic ischaemic stroke patients with fatigue assessment.  No TIA, ICH, death at timepoint or unable to visit hospital for both timepoints. | Overall not reported | 63.9% | 6-8 weeks and 1.5yrs FU | CIS | Total score ≥35 | Brain Atrophy, Lesion Lateralisation, Lesion Location, White Matter Hyperintensities |
| Stein et al., 1996 | 189 | Participants aged between 50-85 with unilateral CVA (ischaemic or embolic), recruited as part of a study assessing post-stroke depression.  No psychiatric history, neurological disease or substance abuse. Non-English speakers were excluded. | 67 (11.8) | 48% | 4+ weeks (Mean= 37) | BDI/HDRS | Subscale of BDI (not specified), Subscale of HDRS (not specified) | Lesion Lateralisation |
| Tang et al., 2010 | 334 | Chinese patients with first ever or recurrent stroke (clinically, CT and MRI confirmed), aged 18+.  Participants must be Cantonese speaking and have had a stroke within 7 days of admission.  No TIA, haemorrhage, subdural haematoma or SAH. No history of central nervous system disease, narcolepsy, sleep apnoea, hypothyroidism, cognitive impairment, aphasia or depression. | Overall not reported | 65.3% | 3M | FSS | Average score of ≥ 4.0 | Lesion Location, Lesion Volume, Number of Infarcts |
| **Study** | **Sample Size** | **Population** | **Age (M (SD))** | **Gender (% male)** | **Time since Stroke** | **Primary Fatigue Measure** | **How is Fatigue Categorised?** | **MRI variables investigated** |
| Tang et al., 2013 | 500 | Chinese patients with first ever or recurrent stroke (clinically and MRI confirmed), aged 18+.  Participants must be Cantonese speaking and have had a stroke within 7 days of admission.  No TIA, haemorrhage, subdural haematoma or SAH. No history of central nervous system disease, cancer, narcolepsy, sleep apnoea, hypothyroidism, cognitive impairment, dementia, aphasia, visual or auditory impairment, depression, recurrent stroke within 3-month follow-up or frailty. | Overall not reported | 64.8% | 3M | FSS | Average score of ≥ 4.0 | Lesion Location, Lesion Volume, Number of Infarcts, White Matter Hyperintensities |
| Tang, Liu et al., 2014 | 199 | Chinese, Cantonese-speaking stroke patients with cerebral microbleeds and MRI scan.  No neurological or comorbid disease, recurrent stroke before 3-month follow-up, severe aphasia, auditory or visual impairment, dementia, depression, alcoholism or physical frailty. | Overall not reported | 70.35% | 3M | FSS | Average score of ≥ 4.0 | Cerebral Microbleeds, Lesion Location, Lesion Volume, Number of Infarcts, White Matter Hyperintensities |
| Tang, Chen et al., 2014 | 435 total (97 included) | Chinese patients with first ever or recurrent stroke (clinically and CT confirmed), aged 18+.  Participants must be Cantonese speaking and have had a stroke within 7 days of admission.  No TIA, haemorrhage, subdural haematoma or SAH. No history of central nervous system disease, cognitive impairment, dementia, aphasia, visual or auditory impairment, depression, psychiatric disorder or recurrent stroke within 3-month follow-up. | Overall not reported | 47.4% | 3M and 15M | FSS | Average score of ≥ 4.0 | Lesion Location, Number of Infarcts, Lesion Volume, White Matter Hyperintensities |
| Tynterova et al., 2022 | 80 | Confirmed unilateral ischaemic stroke in carotid system, aged 40-70, ability to give informed consent, admitted to hospital within 24hrs of CVA onset, NIHSS score of $\leq$15, consciousness at time of assessment and ability to answer questions.  No history of severe cognitive impairment or mental illness, vertebrobasilar stroke, ASPECT score of $\leq$7 for ischaemic stroke or any decompensated comorbidities.. | Overall not reported | 48.75% | 2-3 days post-stroke | MFI (General, physical, or mental subscales) | Score of 12+ on subscales | Lesion Lateralisation |
| **Study** | **Sample Size** | **Population** | **Age (M (SD))** | **Gender (% male)** | **Time since Stroke** | **Primary Fatigue Measure** | **How is Fatigue Categorised?** | **MRI variables investigated** |
| Ulrichsen et al., 2021 | 84 | Ischaemic or haemorrhagic stroke patients in chronic phase (≥3 months), 18+.  No contraindication to MRI and no neurological disease. | 65.8 (12.6) | 71.4% | ≥3 months since admission | FSS | Average score of ≥ 4.0 | Lesion Lateralisation, Lesion Volume, Network Measures |
| Van Eijsden et al., 2012 | 250 total (242 included) | Patients with stroke as defined by WHO criteria, 18+, who had been discharged home from rehabilitation centre.  Participants had to live within 30km of rehabilitation centre and needed to be able to walk 10m without assistance to be included.  No cognitive or communication impairments. | 57.1 (10.3) | 64.9% | Mean (SD) days: 97 (46.9) | FSS | Average score of ≥ 4.0 | Lesion Lateralisation |
| Visser, Goodin et al., 2019 | 28 | Patients aged 18+ with ischaemic stroke more than 3 months ago and score of ≥60 on MFI-20 across all domains.  No contraindications to modafinil, history of neuropsychiatric disease and other known causes of fatigue, no contraindications to MRI and no claustrophobia. | 62 (14.3) | 64% | >3 months | MFI-20 | Continuous score. Higher scores = higher fatigue. | Network Measures |
| Visser, Maréchal et al., 2019 | 28 total (23 included) | Patients aged 18+ with ischaemic stroke more than 3 months ago and score of ≥60 on MFI-20 across all domains.  No contraindications to modafinil, history of neuropsychiatric disease and other known causes of fatigue, contraindications to MRI and claustrophobia. | 63.2 (15.35) | 56.5% | >3 months | MFI-20 | Continuous score. Higher scores = higher fatigue. | Network Measures |
| Wang et al., 2014 | 265 | First ever acute ischaemic stroke patients (<2 weeks).  Patients in intensive care were excluded. | 63 (12.1) | 57% | <2 weeks | FSS | Average score of ≥ 4.0 | Lesion Lateralisation |
| Wang et al., 2022 | 361 | Individuals 18+ with first ever acute ischaemic stroke confirmed on MRI and willingness and ability to participate.  No inability to carry out testing, cancer or other chronic disease, pre-existing neurological disease, history of fatigue or other mental disease prior to stroke, those on antidepressants, contraindication to MRI or those with poor cognitive ability/ MMSE $\leq$ 10. | Overall not reported | 63.72% | Mdn: 13 days | FSS | Average score of ≥ 4.0, average scores also used as continuous variable (higher scores= higher fatigue). | Lesion Lateralisation, Lesion Volume |
| **Study** | **Sample Size** | **Population** | **Age (M (SD))** | **Gender (% male)** | **Time since Stroke** | **Primary Fatigue Measure** | **How is Fatigue Categorised?** | **MRI variables investigated** |
| Zhang et al., 2021 | 40 (16 with fMRI) | Mild ischaemic stroke patients within 2 weeks of stroke (+/- 2 days)  No NIHSS ≥4, cognitive impairment, alcohol or drug abuse, fatigue prior to stroke, long-term medication use for mental health, nervous system or thyroid disease, depression, inability to complete follow-ups and difficulties in consciousness, understanding, hearing, vision or language that prevented participation. | 59.3 (10.7) | 85% | 14 days +/- 2 days | FSS | Total score of ≥36 | Lesion Lateralisation, Lesion Location, Network Measures |
| Zhang et al., 2023 | 230 | Minor ischaemic stroke (NIHSS <4 within 7 days of onset), patients between 40-70 and cognitive ability to take part.  No central nervous system disease other than stroke, contraindication to MRI, significant dysarthria or aphasia, dementia (MMSE= <17), recurrent stroke within 3 months of initial stroke, severe heart/lung/kidney/ liver conditions or malignant tumours. | Overall not reported | 60.87% | Acute stroke | FSS | Average score of ≥ 4.0 | Lesion Location |
| *Note.*  *n* = 6,369. * = . Mdn (IQR). † = analyses do not include TIA but only overall ages including TIA patients given.  BDI = Beck’s Depression Inventory, CIS = Checklist Individual Strength, FACIT = Functional Assessment of Chronic Illness Therapy, FAI = Fatigue Assessment Inventory, FAS = Fatigue Assessment Scale, FIS = Fatigue Impact Scale, FSMC= Fatigue Scale for Motor and Cognitive Functions, FSS = Fatigue Severity Scale, HDRS = Hamilton Depression Rating Scale, MFI = multidimensional fatigue inventory, NIHSS = National Institute of Health Stroke Scale, PHQ = Patient Health Questionnaire, VAS = visual analogue scale. | | | | | | | | |

**sTable 3. Table to show characteristics and findings of studies assessing lesion location.**

| sTable 3. Table to show characteristics and findings of studies assessing lesion location. | | | |
| --- | --- | --- | --- |
| ***Study*** | ***n*** | ***Lesion Location*** | ***Findings*** |
| **Cortical** | | | |
| Chen et al., 2015 | 218 | Cortical region | There was no correlation between PSF and cortical lesions in total population (*p* = 0.20) or those without post-stroke depression (*p* = 0.20). |
| Choi-Kwon et al., 2005 | 220 | Cortex | Presence of PSF was not related to cortex lesions, even when subcategorised as frontal, occipital, parietal and temporal regions. |
| Hubacher et al., 2012* | 31 | Cortical | There was a small effect size for cortical lesions having higher cognitive fatigue (d = 0.21). |
| Radman et al., 2012 | 109 | Cortical | There was no correlation between fatigue score and cortical lesions. There was a trend for left parietal lesions (*p =* 0.086). |
| Tang et al., 2010 | 334 | Cortical | Presence of PSF was not related to cortical lesions subcategorised as frontal (*p* = 0.241), occipital (*p* = 0.704), parietal (*p* = 0.792) and temporal (*p* = 0.204) regions. |
| Tang et al., 2013 | 500 | Cortical | Presence of PSF was not related to cortical lesions subcategorised as frontal (*p* = 0.122), occipital (*p* = 1.00), parietal (*p* = 0.816) and temporal (*p* = 0.201) regions. |
| Tang, Liu et al., 2014 | 199 | Cerebral cortex | There was no difference between the fatigued group and the non-fatigued group in number of cerebral cortex lesions (*p* = 0.331) |
| Tang, Chen et al., 2014 | 97 | Cortical | Presence of PSF was not related to cortical lesions (*p* = 0.88) or when subcategorised as frontal (*p* = 0.17), occipital (*p* = 0.232), parietal (*p* = 1.00) and temporal (*p* = 1.00) regions. |
| Zhang et al., 2021 | 40 | Cortical | Presence of PSF was not related to cortical lesions subcategorised as frontal (*p* = 0.72), occipital (*p* = 0.168), parietal (*p* = 0.72) and temporal (*p* = 0.536) regions. |
| I**nsula** | | | |
| Manes, 1999* | 25 | Insula | There was a significant difference in subjective anergia and underactivity (*p* = 0.002), as well as tiredness (*p* <0.002), between right insula lesions and non-insula lesions. There was higher frequency of anergia and tiredness in insula lesions compared to cortical lesions. |
| Zhang et al., 2021 | 40 | Insula | There was no difference between the fatigued group and the non-fatigued group in number of insula lesions (*p* = 0.27) |
| **Subcortical** | | | |
| Choi-Kwon et al., 2005 | 220 | Subcortex | Presence of PSF was not related to subcortex lesions. |
| Delva & Delva, 2017* | 107 | Subcortical | Subcortical infarcts were associated with: increased risk of physical PSF at 1 month (OR= 3.15 95% CI [1.26, 7.86], *p* = 0.01) and at 3 months (OR = 2.56 95% CI [1.07, 6.15], *p* = 0.04), and increased risk of activity-related PSF at 1 month (OR= 2.96 95% CI [1.17, 7.51], *p* = 0.02) and 6 months (OR= 2.71 95% CI [1.12, 6.58], *p* = 0.03). |
| Hubacher et al., 2012* | 31 | Subcortical | There was a small effect size for subcortical having higher motor fatigue (d = -0.23) on FSMC. There was a medium effect of subcortical lesions showing higher cognitive fatigue (d = -0.74) and total fatigue (d= -0.53) on MFI-20. |
| Radman et al., 2012 | 109 | Subcortical | There was no correlation between fatigue score and subcortical lesions. |
| **Basal Ganglia** | | | |
| Chen et al., 2015 | 218 | Basal Ganglia and Thalami | There was no correlation between PSF and basal ganglia and thalami lesions in the whole population (*p* = 0.62) or in those without post-stroke depression (*p =* 0.87). |
| Tang et al., 2010* | 334 | Basal Ganglia | The fatigued group had a higher number of basal ganglia lesions compared to the non-fatigued group (*p* = 0.018). Acute basal ganglia infarcts were a significant independent predictor of PSF (OR= 2.08, 95% CI [1.16, 3.75], *p* = 0.014), while controlling for sex, depression score, instrumental activities of daily living and number of acute infarcts. |
| Tang, Liu et al., 2014 | 199 | Basal Ganglia | The was no difference between the fatigued group and the non-fatigued group in number of basal ganglia lesions (*p* = 0.669) |
| Tang, Chen et al., 2014 | 97 | Basal Ganglia | The was no difference between the fatigued group and the non-fatigued group in number of basal ganglia lesions (*p* = 0.684) |
| **Thalamus** | | | |
| Choi-Kwon et al., 2005 | 220 | Thalamus | Presence of PSF was not related to thalamus lesions. |
| Mutai et al., 2017* | 101 | Thalamus | Thalamus and/or brainstem lesions were a significant predictor of general fatigue ($\beta$ = 0.51, *p* < 0.001) while controlling for lesion side and anxiety. |
| Tang et al., 2010 | 334 | Thalamus | The was no difference between the fatigued group and the non-fatigued group in number of thalamus lesions (*p* = 0.885) |
| Tang et al., 2013 | 500 | Thalamus | The was no difference between the fatigued group and the non-fatigued group in number of thalamus lesions (*p* = 0.927). |
| Tang, Liu et al., 2014 | 199 | Thalamus | The was no difference between the fatigued group and the non-fatigued group in number of thalamus lesions (*p* = 1.00) |
| Tang, Chen et al., 2014 | 97 | Thalamus | The was no difference between the fatigued group and the non-fatigued group in number of thalamus lesions (*p* = 1.00) |
| Wang et al., 2022* | 361 | Thalamus | There were no significant lesion-behavioural characteristics in those with or without fatigue, nor with continuous fatigue severity score at baseline.  At follow up (*n* = 324) there was a significant association between a cluster of voxels in the right thalamus and fatigue outcome (Y/N) as well as severity of fatigue. When those with left lesions were flipped along the mid-sagittal line the association between thalamus lesions and fatigue outcome at follow-up (Y/N) persisted.  Those with right thalamus lesions showed higher rates of binary fatigue (p < 0.001) and fatigue severity (p =0.023) when compared to all other lesions. Multivariate analyses showed right thalamus lesions were a predictor of fatigue at follow-up even when controlling for age, sex, lesion volume, hypertension, hypercholesterolaemia, BMI, diabetes, smoking, drinking, NIHSS score, stroke classification, depression and Lubben social score (OR: 2.67, 95% CI 1.46– 4.88). |
| Zhang et al., 2023* | 230 | Thalamus, | There was a significant association between PSF and brainstem, cerebellum, and thalamus lesions (*p* = 0.032). On logistic regression controlling for sex, anxiety, depression, cognitive impairment, sleep disorders and blood biomarkers, lesion location was not associated with fatigue. |
| **Other Subcortical Areas** | | | |
| Appelros, 2006 | 253 | Lacunar Infarct | PSF was not associated with lacunar infarcts (OR: 1.4, 95% CI [0.81, 2.4], *p* = 0.23). |
| Choi-Kwon et al., 2005 | 220 | Lenticulo-capsular area | Presence of PSF was not related to lenticulo-capsular area lesions. |
| Tang et al., 2010* | 334 | Internal Capsule | The fatigued group had a higher number of internal capsule lesions compared to the non-fatigued group (*p* = 0.027) |
| Tang et al., 2013* | 500 | Caudate | The fatigued group had a higher number of caudate lesions compared to the non-fatigued group (*p* = 0.001). Acute infarcts in the caudate (OR= 6.46, 95% CI [2.06, 20.02], *p* = 0.001) were associated with an increased risk of PSF when controlling for sex, depression score, hyperlipidaemia, Barthel index score and acute infarcts in the pons and putamen. |
| Tang et al., 2013 | 500 | Putamen | The fatigued group had a higher number of putamen lesions compared to the non-fatigued group (*p* = 0.043). There was no significant association of putamen infarcts to PSF on multivariate analyses controlling for sex, depression score, hyperlipidaemia, Barthel index score and acute infarcts in the caudate and pons (*p* = 0.338). |
| Tang et al., 2013 | 500 | Globus Pallidus | The was no difference between the fatigued group and the non-fatigued group in number of globus pallidus lesions (*p* = 1.00) |
| Zhang et al., 2021 | 40 | Striatum-thalamus-frontal cortex | There was a trend for a higher number of striatum-thalamus-frontal cortex lesions in the fatigued group compared to the non-fatigued group (*p* = 0.056). |
| **Infratentorial** | | | |
| Chen et al., 2015 | 218 | Infratentorial | There was no correlation between PSF and infratentorial lesions in the whole population (*p* = 0.68) or those without post-stroke depression (*p* = 0.56). |
| Delva & Delva, 2017* | 107 | Infratentorial | Infratentorial lesions were associated with increased risk of global PSF at 3 months (OR: 2.91, 95% CI [1.24, 6.83], *p =* 0.01) and at 6 months (OR: 3.19, 95% CI [1.34, 7.58], *p =* 0.01). |
| Snaphaan et al., 2011* | 108 | Infratentorial | There was a significant difference in infratentorial lesions in those with and without fatigue at baseline (*p =* 0.03), but not at follow up (*p* =0.34). Infratentorial infarcts were associated with increased risk of PSF at baseline (OR: 4.10, 95% CI 1.04, 16.12) but not at follow up (OR 1.78, 95% CI 0.48-6.56) while controlling for age and sex. On multivariate analyses infratentorial lesions were associated to PSF at baseline (OR = 4.69, 95% CI [1.03, 21.47]), while controlling for age, sex, anxiety, depressive symptoms and disability.  There was no difference in prevalence of no fatigue, persistent fatigue, incident fatigue and recovery fatigue (*p =* 0.18). |
| Choi-Kwon et al., 2005 | 220 | Infratentorial v Supratentorial | Presence of PSF did not differ between infratentorial and supratentorial strokes. |
| Liu et al., 2015 | 30 | Infratentorial v Supratentorial | No difference in between low-moderate fatigue and severe fatigue in number of supratentorial and infratentorial lesions (*p =* 0.586). |
| Ormstad et al., 2011 | 34 | Infratentorial v Supratentorial | Not assessed due to small infratentorial sample size (n=4). |
| Tang, Liu et al., 2014 | 199 | Infratentorial | The was no difference between the fatigued group and the non-fatigued group in number of infratentorial lesions (*p* = 0.179) |
| Tang, Chen et al., 2014 | 97 | Infratentorial | The was no difference between the fatigued group and the non-fatigued group in number of infratentorial lesions (*p* = 0.107) |
| **Brainstem** | | | |
| Choi-Kwon et al., 2005 | 220 | Brainstem | Presence of PSF was not related to brainstem lesions. |
| Mutai et al., 2017* | 101 | Brainstem | Thalamus and/or brainstem lesions were a significant predictor of general fatigue ($\beta$ = 0.51, *p* < 0.001) while controlling for lesion side and anxiety. |
| Radman et al., 2012 | 109 | Brainstem | There was no correlation between fatigue score and brainstem lesions. |
| Tang et al., 2010* | 334 | Brainstem | The fatigued group had fewer brainstem lesions compared to the non-fatigued group (*p* = 0.032) |
| Tang, Chen et al., 2014 | 97 | Brainstem | The was no difference between the fatigued group and the non-fatigued group in brainstem lesions (*p* = 0.516) |
| Zhang et al., 2021 | 40 | Brainstem | The was no difference between the fatigued group and the non-fatigued group in number of brainstem lesions (*p* = 0.301) |
| Zhang et al., 2023* | 230 | Brainstem | There was a significant association between PSF and brainstem, cerebellum, and thalamus lesions (*p* = 0.032). On logistic regression controlling for sex, anxiety, depression, cognitive impairment, sleep disorders and blood biomarkers, lesion location was not associated with fatigue. |
| **Cerebellum** | | | |
| Choi-Kwon et al., 2005 | 220 | Cerebellum | Presence of PSF was not related to cerebellar lesions. |
| Radman et al., 2012 | 109 | Cerebellum | There was no correlation between fatigue score and cerebellar lesions. |
| Tang et al., 2010* | 334 | Cerebellum | The fatigued group had fewer cerebellum lesions compared to the non-fatigued group (*p* = 0.022) |
| Tang et al., 2013 | 500 | Cerebellum | The was no difference between the fatigued group and the non-fatigued group in number of cerebellum lesions (*p* = 0.178) |
| Tang, Chen et al., 2014 | 97 | Cerebellum | The was no difference between the fatigued group and the non-fatigued group in number of cerebellum lesions (*p* = 0.195) |
| Zhang et al., 2021 | 40 | Cerebellum | The was no difference between the fatigued group and the non-fatigued group in number of cerebellum lesions (*p* = 0.27) |
| Zhang et al., 2023* | 230 | Cerebellum | There was a significant association between PSF and brainstem, cerebellum, and thalamus lesions (*p* = 0.032). On logistic regression controlling for sex, anxiety, depression, cognitive impairment, sleep disorders and blood biomarkers, lesion location was not associated with fatigue. |
| **Midbrain** | | | |
| Choi-Kwon et al., 2005 | 220 | Midbrain | Presence of PSF was not related to midbrain lesions. |
| Tang et al., 2013 | 500 | Midbrain | The was no difference between the fatigued group and the non-fatigued group in number of midbrain lesions (*p* = 0.686) |
| **Pons** | | | |
| Choi-Kwon et al., 2005 | 220 | Pons | Presence of PSF was not related to pons lesions. |
| Tang et al., 2013* | 500 | Pons | There were significantly fewer pons lesions in the fatigued group compared to the non-fatigues group (*p* = 0.038). Pons infarcts were associated with a reduced risk of PSF (OR= 0.47, 95% CI [0.26, 0.88], *p* = 0.017), when controlling for sex, depression score, hyperlipidaemia, Barthel index score and acute infarcts in the caudate and putamen. |
| **Medulla** | | | |
| Choi-Kwon et al., 2005 | 220 | Medulla | Presence of PSF was not related to medulla lesions. |
| Tang et al., 2013 | 500 | Medulla | The was no difference between the fatigued group and the non-fatigued group in number of medulla lesions (*p* = 0.464) |
| **Subcortical and Cortical** | | | |
| Delva & Delva, 2017 | 107 | Cortical-Subcortical | There were no significant relationships between PSF and cortical-subcortical lesions. |
| Radman et al., 2012 | 109 | Cortical- subcortical | There was no correlation between fatigue score and cortical-subcortical lesions. |
| Hubacher et al., 2012 | 31 | Cortical and Subcortical localisation | No significant association with fatigue on MFI or on FSMC. |
| Snaphaan et al., 2011 | 108 | Both Cortical and Subcortical | There was no difference in prevalence of fatigue at baseline (*p* = 0.07) and follow up (*p =* 0.44) across cortical, subcortical or both lesions nor in prevalence of no fatigue, persistent fatigue, incident fatigue and recovery fatigue (*p =* 0.39) |
| **Other Areas** | | | |
| Becker et al., 2015 | 39 | Infarct Location | There was no association of PSF to lesion location (*p* value not reported). |
| *Note. n* = 3036, * = significant finding. | | | |

**sTable 4. Table to show characteristics and findings of studies assessing lesion volume.**

| sTable 4. Table to show characteristics and findings of studies assessing lesion volume.. | | | | | |
| --- | --- | --- | --- | --- | --- |
| **Author** | **N** | **Study design** | **Population** | **How is lesion volume operationalised?** | **Findings** |
| Becker et al., 2015 | 39 | Cross-sectional | Ischaemic stroke patients | Infarct volume (mm^3^) | No significant association between lesion volume and fatigue (*p* >0.20) |
| Chen et al., 2015 | 218 | Cross-sectional | Acute first or recurrent ischaemic stroke with fatigue | Infarct volume (not specified) | No significant association between infarct volume and fatigue in whole cohort (*r =* -0.03, *p =* 0.66) or in those without post-stroke depression (n = 190, *r =* -0.08, *p* = 0.30). |
| Chen & Marsh, 2018 | 203 | Prospective Cohort study | Acute first or recurrent ischaemic stroke | Infarct volume (cc) | There was no significant association between infarct volume and fatigue at subacute stage (*p =* 0.112) and the chronic stage (*p=* 0.167) on univariate linear regression. |
| Choi Kwon et al., 2005 | 220 | Cross-sectional | Ischaemic or Haemorrhagic Stroke patients | Large or small ($\leq$2cm) | Post stroke fatigue was not related to lesion size. |
| Cotter et al., 2022 | 63 | Cohort Study | Ischaemic stroke patients | Lesion volume (ml) | There was no significant difference between lesion volumes of those with and without fatigue (*p* = 0.534) with an effect size of 0.09. |
| Delva & Delva, 2017 | 107 | Cross-sectional | Acute ischaemic stroke patients. | Infarct volume (cm^3^) | No significant association between infarct volume and fatigue at any time point (acute, 1 month, 3 months, 6 months). |
| Marsh et al., 2018 | 151 | Prospective Cohort study | Minor Stroke Patients (NIHSS <4) | Stroke volume (cc) | There was no significant association of stroke volume to FACIT scores (*p* = 0.84) |
| Ormstad et al., 2011 | 34 | Cross-sectional | First Ischaemic or Haemorrhagic Stroke patients | Infarct volume (cm^3^) | There was no significant correlation between infarct volume and FSS score at 6 months (*p* = 0.313), 12 months (*p* = 0.405) and 18 months (*p* =0.927) |
| Pihlaja et al., 2014 | 133 | Cross-sectional | First ever Supratentorial Ischemic stroke | Infarct size:  Small <1.5cm  Medium 1.5-4.0cm  Large >4.0cm | There was no significant difference between those with and without post-stroke fatigue in infarct size (*p =* 0.818) |
| Schaechter et al., 2023 | 12 | Cross-sectional | Unilateral ischaemic stroke in the middle cerebral artery | Lesion volume (cm^3^) | There was no significant correlation between lesion volume and the general fatigue subscale of the MFI-GF (Kendall’s $\tau$ = 0.37, *p* = 0.11). |
| Tang et al., 2010 | 334 | Prospective Cohort Study | Acute first ischaemic stroke | Size of acute Infarct (cm^3^) | There was no significant difference between those with and without post-stroke fatigue in acute infarct size (*p =* 0.876) |
| Tang et al., 2013 | 500 | Prospective Cohort Study | Acute first ischaemic stroke | Volume of acute Infarcts (cm^3^) | There was no significant difference between those with and without post-stroke fatigue in volume of all acute infarcts (*p =* 0.767) |
| Tang, Liu et al., 2014 | 199 | Prospective Cohort Study | Acute first ischaemic stroke | Volume of acute infarcts (cm^3^) | There was no significant difference between those with and without post-stroke fatigue in acute infarct size (*p =* 0.943) |
| Tang, Chen et al., 2014 | 97 | Prospective Cohort Study | Acute first ischaemic stroke | Volume of acute infarct (cm^3^) | There was no significant difference between those with and without post-stroke fatigue in acute infarct size (*p =* 0.777) |
| Ulrichsen et al., 2021 | 84 | Cross-sectional | Chronic ischaemic or haemorrhagic stroke | Number of voxels lesioned / number of voxels in a lesion | There was no significant correlation between fatigue and number of voxels lesioned (BF = 0.28, *δ*  = 0.05, 95% CI [-0.16, 0.25] or number of voxels in a lesion (BF = 0.29, *δ*  = 0.05, 95% CI [-0.16, 0.26]. |
| Wang et al., 2022 | 361 | Prospective Cohort Study | Acute first ischaemic stroke | Lesion volume (ml) | There was no significant difference between lesion volumes of those with and without stroke at baseline (*p* = 0.587) or at follow up (*p* = 0.112). |
| *Note. n* = 2755. | | | | | |

**sTable 5. Table to show characteristics and findings of studies assessing brain atrophy.**

| sTable 5. Table to show characteristics and findings of studies assessing brain atrophy. | | | | | |
| --- | --- | --- | --- | --- | --- |
| **Author** | **N** | **Study design** | **Population** | **How is brain atrophy operationalised?** | **Findings** |
| Chen et al., 2015 | 218 | Cross-sectional | Acute first or recurrent ischaemic stroke | Ventricle to brain ratio (VBR) | No significant correlation between VBR and fatigue score in the whole cohort (*r* = 0.10, p = 0.16) and those without post-stroke depression (n = 190, *r* = 0.11, *p* = 0.15). |
| Delva & Delva, 2017 | 107 | Cross-sectional | Acute ischaemic stroke | - bifrontal index (BFI) - bicaudate index (BCI) - maximum diameter of the third ventricle (mm) - cortical atrophy index (CAI) | There was no significant association between any of the brain atrophy measures and any fatigue subscale at any timepoint (acute, 1 month, 3 months and 6 months). |
| Kutlubaev et al., 2013 | 107 | Longitudinal cohort study | Ischaemic or Haemorrhagic Stroke patients | - Any atrophy - Atrophy in the: Cerebellum, Brainstem, Frontal, Parietal, Temporal, Occipital lobes (subdivided into left and right and further subdivided into cortical/subcortical) | There was no significant relationship between FAS score and: central and cortical brain atrophy (*p* = 0.73) or atrophy in any of the listed regions. |
| Snaphaan et al., 2011 | 108 | Cohort study | Ischaemic stroke patients | - Cortical atrophy ratio (CAR) - Subcortical atrophy ratio (SAR) | There was no significant difference in CAR (*p* = 0.28) or SAR (*p* = 0.10) across those with no fatigue, persistent fatigue, incident fatigue and recovery fatigue. |
| *Note. n =* 540 | | | | | |

| **sTable 6. Table to show characteristics and findings of studies assessing number of infarcts (both acute and old).** | | | | | |
| --- | --- | --- | --- | --- | --- |
| sTable 6. Table to show characteristics and findings of studies assessing number of infarcts (both acute and old). | | | | | |
| **Number of Old Infarcts** | | | | | |
| **Author** | **N** | **Study design** | **Population** | **How are old infarcts operationalised?** | **Findings** |
| Chen et al., 2015 | 218 | Cross-sectional | Acute first or recurrent ischaemic stroke | Total number of old infarcts on diffusion weighted imaging (DWI) | There was no significant correlation between number of old infarcts and fatigue score in the whole cohort (*r =* -0.02, *p* = 0.83) and those without post-stroke depression (n = 190, *r* = -0.03, *p =* 0.67). |
| Kutlubaev et al., 2013 | 107 | Longitudinal cohort study | Ischaemic or Haemorrhagic Stroke patients | Presence of old vascular lesions | There was no association between fatigue and presence of old vascular lesions (*p* = 0.49) |
| Tang et al., 2013 | 500 | Prospective Cohort Study | First acute ischaemic stroke | Total number of old infarcts assessed by neurologist | There was no significant difference between those with and without fatigue in the number of old infarcts (*p =* 0.124). |
| Tang, Chen et al., 2014 | 97 | Prospective Cohort Study | First acute ischaemic stroke with fatigue | Total number of old infarcts assessed by neurologist on DWI | There was no significant difference between remitters (recovered fatigue) and non-remitters (persistent fatigue) in the number of old infarcts (*p =* 0.37). |
| **Number of Acute Infarcts** | | | | | |
| **Author** |  | **Study design** | **Population** | **How are acute infarcts operationalised?** | **Findings** |
| Tang et al., 2010 | 334 | Prospective Cohort Study | First acute ischaemic stroke | Total number of acute infarcts assessed by neurologist | Those with fatigue had a higher number of acute infarcts compared to those without fatigue infarcts *p* <0.034. On multivariate analyses controlling for acute infarcts in the basal ganglia, gender, depression score and instrumental activities of daily living, acute infarct number was not a predictor of PSF (OR = 1.05 95% CI [0.94, 1.17], *p* = 0.39. |
| Tang et al., 2013 | 500 | Prospective Cohort Study | First acute ischaemic stroke | Total number of acute infarcts assessed by neurologist | There was no significant difference between those with and without fatigue in the number of acute infarcts (*p =* 0.10). |
| Tang, Liu et al., 2014 | 199 | Prospective Cohort Study | First acute ischaemic stroke with fatigue | Total number of acute infarcts | There was no significant difference between those with and without fatigue in the number of acute infarcts (*p =* 0.794). |
| Tang, Chen et al., 2014 | 97 | Prospective Cohort Study | First acute ischaemic stroke with fatigue | Total number of acute infarcts by neurologist on DWI | There was no significant difference between remitters (recovered fatigue) and non-remitters (persistent fatigue) in the number of acute infarcts (*p =* 0.13). |
| *Note.* Number of old infarcts (*n* = 922), number of acute infarcts (*n* = 1130) | | | | | |

**sTable 7. Table to show characteristics and findings of studies assessing white matter hyperintensities.**

| sTable 7. Table to show characteristics and findings of studies assessing white matter hyperintensities. | | | | | |
| --- | --- | --- | --- | --- | --- |
| **Author** | **N** | **Study design** | **Population** | **How are white matter hyperintensities operationalised?** | **Findings** |
| Chen et al., 2015 | 218 | Cross-sectional | Acute first or recurrent ischaemic stroke | Deep White Matter hyperintensities (DWMH) and Periventricular hyperintensities (PVH) assessed with Fazekas Scale score | Spearman’s correlation showed no significant association between PVH score and fatigue score (*r* = 0.06, *p* = 0.40), or DWMH score and fatigue score (*r* = 0.08, *p =* 0.22). Spearman’s correlation also no showed association between the above variables when excluding participants with post-stroke depression (n = 190, PVH score: *r* = 0.08, *p* = 0.27, DWMH score:  *r* = 0.08, *p* = 0.25). |
| Delva & Delva, 2017 | 107 | Cross-sectional | Acute ischaemic stroke patients | Overall Fazekas Scale score (sum of individual DWMH and PVH scores). | A summed Fazekas score of 1 was a significant predictor of mental fatigue at both 3 months (OR: 1.54 [95% CI 1.05, 2.21], *p* = 0.03) and at 6 months (OR: 1.79 [95% CI 1.20, 2.65], *p* = 0.04). |
| Kutlubaev et al., 2013 | 107 | Longitudinal cohort study | Ischaemic and haemorrhagic stroke patients | Categorised as none, mild, moderate and severe WMH by an experienced neuroradiologist. | There was no significant association between FAS score and central and subcortical WML (*p* = 0.78).  There was also no significant correlation between FAS score and WMHs in: frontal, temporal, occipital and parietal lobe (subdivided into left right and further subdivided in central and subcortical regions and assessed separately). In addition, there was no significant correlation between FAS score and WMHs in the left and right brainstem. |
| Snaphaan et al., 2011 | 108 | Cohort study | Ischaemic stroke patients | Age related white matter changes (ARWMC) scale score | There was no significant difference in ARWMC score between those with and without fatigue at baseline (*p* = 0.10) and at follow up (*p =* 0.17) whilst controlling for age. There was also no significant difference in ARWMC score between those with no fatigue, persistent fatigue, incident fatigue and recovery fatigue (*p* = 0.07). |
| Tang et al., 2013 | 500 | Prospective Cohort Study | First acute ischaemic stroke | Deep White Matter hyperintensities (DWMH) and Periventricular hyperintensities (PVH) assessed with Fazekas Scale score | No significant difference between fatigued and non-fatigued participants in DWMH Fazekas score (*p =* 0.338) and PVH Fazekas score (*p* = 0.728). |
| Tang, Liu et al., 2014 | 199 | Prospective Cohort Study | First acute ischaemic stroke and post-stroke fatigue | Deep White Matter hyperintensities (DWMH) and Periventricular hyperintensities (PVH) assessed with Fazekas Scale score | No significant difference between fatigued and non-fatigued participants in DWMH Fazekas score (*p =* 0.935) and PVH Fazekas score (*p* = 0.980). |
| Tang, Chen et al., 2014 | 97 | Prospective Cohort Study | First acute ischaemic stroke and post-stroke fatigue | Deep White Matter hyperintensities (DWMH) and Periventricular hyperintensities (PVH) assessed with Fazekas Scale score | No significant difference between non-remitters (fatigued) and remitters (recovered fatigue) in DWMH Fazekas score (*p =* 0.549) and PVH Fazekas score (*p* = 0.637). |
| Note. *n =* 1336. * = Mdn (IQR). | | | | | |

sTable 8. Table to show characteristics of studies included in each meta-analysis.

| sTable 8. Table to show characteristics of studies included in each meta-analysis | | | | |  |
| --- | --- | --- | --- | --- | --- |
| Study | Neuroimaging measure used for MA | Fatigue Measure used for MA | Timepoint Assessed in MA | Controlled for confounders? (Age and sex) | Risk of Bias score |
| ***Lesion Lateralisation*** | | | | |  |
| Appelros, 2006 | Odds ratio for left-side stroke. | Self-report (fatigue yes/no) | 12 months | N | 5/8 |
| Cotter et al., 2021 | Left vs Right | PHQ-9 (Q4, score >0) | 3 months | N | 5/8 |
| Khan & Delargy, 2017 | Left vs Right | FSS (binarized score cut-off not reported) | <6 months | N | 3/8 |
| Kindred et al., 2023 | Left vs Right | FSS (Total score ≥36 = fatigue) | >6 months | N | 4/8 |
| Kjeverud et al., 2020 | Left vs Right | FSS (Low- Moderate vs severe fatigue groups). | Trajectory groups across acute stage to 18 months | N | 6/8 |
| Liu et al., 2015 | Left vs Right | FSMC (Low- moderate vs severe fatigue groups) | >3 months | N | 3/8 |
| Lynch et al., 2007 | Left vs Right | Self-report in line with case-definition (yes/no) | Mdn (IQR) (days):23 (93-217) | N | 5/8 |
| Miller et al., 2013 | Left vs Right | FSS (average score ≥ 4.0 = fatigue) | >6 months | N | 3/8 |
| Pihlaja et al., 2014 | Left vs Right | Combined score on Profile of Mood States-F and Profile of Moods States-V (scores above 95^th^ percentile = fatigue) | Mean (SD) days 84.7 (23.9) | N | 4/8 |
| Ponchel et al., 2016 | Left vs Right | Chalder Fatigue Scale (score of 4+ = fatigue) | 6 months | N | 4/8 |
| Schaechter et al., 2023 | Left vs Right | MFI- General Fatigue subscale (≥ 12 = fatigue) | 1-3 years post-stroke. (Mean (SD) years: 1.72 (0.43) | N | 3/8 |
| Snaphaan et al., 2011 | Left vs Right | CIS (Total score ≥35 = fatigue) | 6-8 weeks | N | 6/8 |
| Stein et al., 1996 | Left vs Right | Subscale of HDRS (unspecified) | 4+ weeks (Mean= 37) | N | 3/8 |
| Wang et al., 2014 | Left vs Right | FSS (average score ≥ 4.0 = fatigue) | <2 weeks | N | 6/8 |
| Wang et al., 2022 | Left vs Right | FSS (average score ≥ 4.0 = fatigue) | Mdn 13 days | N | 7/8 |
| Zhang et al., 2021 | Left vs Right | FSS (Total score ≥36 = fatigue) | 14 days +/- 2 days | N | 4/8 |
| ***Infratentorial Lesion Location*** | | | | |  |
| Delva & Delva, 2017 | Odds ratio for infratentorial location | Global PSF (score of 12+ = fatigue) | 3 months | N | 4/8 |
| Liu et al., 2015 | Infratentorial vs supratentorial | FSMC (Low- moderate vs severe fatigue groups) | >3 months | N | 3/8 |
| Snaphaan et al., 2011 | Infratentorial vs non-infratentorial | CIS (Total score ≥35 = fatigue) | 6-8 weeks | N | 6/8 |
| Tang, Liu et al., 2014 | Infratentorial vs cerebral cortex, subcortical white matter, basal ganglia or thalamus. | FSS (average score ≥ 4.0 = fatigue) | 3 months | N | 5/8 |
| ***Lesion Volume*** | | | | | |
| Cotter et al., 2021 | Lesion volume (ml) | PHQ-9 (Q4, score >0) | 3 months | N | 5/8 |
| Schaechter et al., 2023 | Infarct volume (cm^3^) | MFI- General Fatigue subscale (≥ 12 = fatigue) | 1-3 years post-stroke. (Mean (SD) years: 1.72 (0.43) | N | 3/8 |
|  |  |  |  |  |  |
|  |  |  |  |  |  |
| ***WMH*** | | | | |  |
| Delva & Delva, 2017 | Odds ratio for Fazekas score 1 | Mental PSF (score of 12+ = fatigue) | 3 months | N | 4/8 |
| Snaphaan et al., 2011 | Mdn (range) on age-related white matter changes scale. Group data converted from Mdn(range) to M(SD) using Hozo et al. 2005.^53^ Then, mean-standard differences re-expressed as Odds ratios as detailed in Cochrane Handbook (Section 10.6).^54^ | CIS (Total score ≥35 = fatigue) | 6-8 weeks | N | 6/8 |
| Tang et al., 2013 | Mean score on Fazekas (deep white matter hyperintensities). Mean-standard differences were re-expressed as ORs as detailed in Cochrane Handbook (Section 10.6).^54^ | FSS (average score ≥ 4.0 = fatigue) | 3 months | N | 6/8 |

| sTable 9. Table to show study aim and whether studies were primary or secondary analyses. | | | | |
| --- | --- | --- | --- | --- |
| **Study** | **Study Aim** | **Secondary or Sub-analyses? (Y/N)** | **Secondary or sub-analyses (specify)** | **Included in meta-analysis?** |
| Appelros, 2006 | To assess predictors and prevalence of post-stroke pain and fatigue. | N |  | Y-Lesion Lateralisation |
| Becker et al., 2015 | To assess biological basis of post-stroke fatigue. | Y | Both. Data collected as part of larger trial, analysis of fatigue and neuroimaging is sub-analysis. | N |
| Chen et al., 2015 | To assess risk factors for post-stroke fatigue and its effect on activities of daily living and quality of life. | N |  | N |
| Chen & Marsh, 2018 | To assess predictors of PSF and how they change over time since stroke | N |  | N |
| Choi-Kwon et al., 2005 | To assess characteristics associated with post-stroke fatigue. | N |  | N |
| Cotter et al., 2022 | To assess functional connectivity in post-stroke fatigue, | N |  | Y-Lesion Lateralisation |
| De Doncker et al., 2021 | To assess the role of corticospinal excitability in post-stroke fatigue. | N |  | N |
| Delva & Delva, 2017 | To assess risk factors of poststroke fatigue and effect on activities of daily living and quality of life | N |  | Y- Infratentorial Lesion Location, White Matter Hyperintensities |
| Delva et al., 2017 | To assess clinical factors associated with post-stroke fatigue at 3 months. | N |  | N |
| Duncan et al., 2015 | To assess whether physical activity is associated with post-stroke fatigue. | Y | Sub-analyses | N |
| Hubacher et al., 2012 | To characterise post-stroke fatigue from the acute to chronic phase. | Y | Sub-analyses | N |
| Jaracz et al., 2007 | To describe the prevalence of fatigue and test its association with physical, psychological and social functioning as well as clinical and demographic factors | N |  | N |
| Khan & Delargy, 2017 | To assess prevalence of, and variables associated with, post-stroke fatigue. | N |  | Y-Lesion Lateralisation |
| Kindred et al., 2023 | To assess role of hemispheric asymmetry in PSF. | N |  | Y-Lesion Lateralisation |
| Kjeverud et al., 2020 | To evaluate the trajectories of fatigue and whether trajectories were associated with sociodemographic and medical symptoms. | N |  | Y-Lesion Lateralisation |
| Kutlubaev et al., 2013 | To assess clinical and CT predictors of fatigue at 1 month. | N |  | N |
| Lamb et al., 2013 | To assess impact of impaired cognition, negative affect and fatigue on cognitive complaints in older adults with stroke. | Y | Sub-analyses | N |
| Liu et al., 2015 | To assesses the role of Th17 and Treg in post-stroke fatigue. | Y | Sub-analyses | Y-Lesion Lateralisation, Infratentorial Lesion Location |
| Lynch et al., 2007 | To develop a case definition of post-stroke fatigue. | Y | Sub-analyses | Y- Lesion Lateralisation |
| Manes, 1999 | To assess neuropsychiatric effects on insular stroke | N |  | N |
| Marsh et al., 2018 | To assess socioeconomic and stroke severity on outcomes after minor stroke. | Y | Sub-analyses | N |
| Miller et al., 2013 | To assess physical/motor symptoms associated with fatigue and pain after stroke. | Y | Sub-analyses | Y- Lesion Lateralisation |
| Mutai et al., 2017 | To assess factors associated with PSF | N |  | N |
| Naess et al., 2005 | To assess factors associated with post-stroke fatigue at long-term follow up | N |  | N |
| Ondobaka et al., 2022 | To evaluate the role of effective connectivity in post-stroke fatigue. | N |  | N |
| Ormstad et al., 2011 | To assess the role of serum cytokine and glucose levels in post-stroke fatigue. | Y | Sub-analyses | N |
| Pihlaja et al., 2014 | To assess association of post-stroke fatigue to cognition and depression after stroke. | Y | Sub-analyses | Y- Lesion Lateralisation |
| Ponchel et al., 2016 | To assess the role of medication on fatigue at 6 months post-stroke. | Y | Sub-analyses | Y- Lesion Lateralisation |
| Radman et al., 2012 | to assess association between fatigue following strokes and poststroke mood, cognitive dysfunction, disability, and infarct | N |  | N |
| Schaechter et al., 2023 | To assess the role of structural and functional connectivity in PSF. | N |  | Y-Lesion Lateralisation |
| Schepers et al., 2006 | To assess the course of post-stroke fatigue and whether it is related to personal and/or stroke characteristics. | N |  | N |
| Snaphaan et al., 2011 | To assess the course of PSF and its risk factors. | N |  | Y-Lesion Lateralisation, Infratentorial Lesion Location, White Matter Hyperintensities |
| Stein et al., 1996 | To evaluate the discriminative properties of somatic and nonsomatic symptoms for post-stroke depression. | Y | Sub-analyses | Y-Lesion Lateralisation |
| Tang et al., 2010 | To assess the role of infarct location in post-stroke fatigue. | N |  | N |
| Tang et al., 2013 | To assess the role of infarct location, namely caudate infarct location, in post-stroke fatigue. | N |  | Y- White Matter Hyperintensities |
| Tang, Liu et al., 2014 | To assess role of cerebral microbleeds in post-stroke fatigue. | N |  | Y- Infratentorial Lesion Location |
| Tang, Chen et al., 2014 | To assess role of infarct location in post-stroke fatigue. | N |  | N |
| Tynterova et al., 2022 | To assess role of lesion lateralisation on neuropsychiatric outcome after acute stroke. | N |  | N |
| Ulrichsen et al., 2021 | To assess whether disconnectivity is associated to post-stroke fatigue. | N |  | N |
| Van Eijsden et al., 2012 | To assess whether personal factors, characteristics of stroke, physical, cognitive, and emotional functions, and activities influence PSF. | Y | Secondary analyses. Data collected in the FIT-STROKE trial. | N |
| Visser, Goodin et al., 2019 | To evaluate the association between modafinil treatment and functional connectivity in post-stroke fatigue. | N |  | N |
| Visser, Maréchal et al., 2019 | To predict modafinil treatment response in post-stroke fatigue using functional connectivity. | N |  |  |
| Wang et al., 2014 | To identify factors that influence fatigue prevalence in the acute phase after first-ever ischemic stroke. | N |  | Y- Lesion Lateralisation |
| Wang et al., 2022 | To assess role of lesion location in VSLM. | N |  | Y- Lesion Lateralisation |
| Zhang et al., 2021 | To assess whether biochemical factors and imaging are associated with post-stroke fatigue in a Chinese population | N |  | Y- Lesion Lateralisation |
| Zhang et al., 2023 | To test whether biomarkers and neuropsychology can predict PSF. | N |  | N |
